# Clinical characterization and placental pathology of mpox infection in hospitalized patients in the Democratic Republic of the Congo

**DOI:** 10.1371/journal.pntd.0010384

**Published:** 2023-04-20

**Authors:** Phillip R. Pittman, James W. Martin, Placide Mbala Kingebeni, Jean-Jacques Muyembe Tamfum, Gaston Mwema, Qingwen Wan, Pierre Ewala, Jules Alonga, Guy Bilulu, Mary G. Reynolds, Xiaofei Quinn, Sarah Norris, Michael B. Townsend, Panayampalli S. Satheshkumar, James Wadding, Bryony Soltis, Anna Honko, Fernando B. Güereña, Lawrence Korman, Kerry Patterson, David A. Schwartz, John W. Huggins, on behalf of The Kole Human Mpox Infection Study Group

**Affiliations:** 1 Division of Medicine, U.S. Army Medical Research Institute of Infectious Diseases (USAMRIID), Fort Detrick, Maryland, United States of America; 2 Walter Reed National Military Medical Center, Bethesda, Maryland, United States of America; 3 Institut National de Recherche Biomédicale, Ministère de la Santé Publique, Kinshasa-Gombe B.P. 1197, Democratic Republic of the Congo (DRC); 4 L’Hôpital Général de Référence de Kole, Kole, Democratic Republic of the Congo; 5 Poxvirus and Rabies Branch, Centers for Disease Control and Prevention, Atlanta, Georgia, United States of America; 6 Perinatal Pathology Consulting, Atlanta, Georgia, United States of America; University of North Carolina at Chapel Hill School of Medicine, UNITED STATES

## Abstract

We describe the results of a prospective observational study of the clinical natural history of human monkeypox (mpox) virus (MPXV) infections at the remote L’Hopital General de Reference de Kole (Kole hospital), the rainforest of the Congo River basin of the Democratic Republic of the Congo (DRC) from March 2007 until August 2011. The research was conducted jointly by the Institute National de Recherche Biomedical (INRB) and the US Army Medical Research Institute of Infectious Diseases (USAMRIID). The Kole hospital was one of the two previous WHO Mpox study sites (1981–1986). The hospital is staffed by a Spanish Order of Catholic Nuns from La Congregation Des Soeurs Missionnaires Du Christ Jesus including two Spanish physicians, who were members of the Order as well, were part of the WHO study on human mpox. Of 244 patients admitted with a clinical diagnosis of MPXV infection, 216 were positive in both the Pan-Orthopox and MPXV specific PCR. The cardinal observations of these 216 patients are summarized in this report. There were three deaths (3/216) among these hospitalized patients; fetal death occurred in 3 of 4 patients who were pregnant at admission, with the placenta of one fetus demonstrating prominent MPXV infection of the chorionic villi. The most common complaints were rash (96.8%), malaise (85.2%), sore throat (78.2%), and lymphadenopathy/adenopathy (57.4%). The most common physical exam findings were mpox rash (99.5%) and lymphadenopathy (98.6%). The single patient without the classic mpox rash had been previously vaccinated against smallpox. Age group of less than 5 years had the highest lesion count. Primary household cases tended to have higher lesion counts than secondary or later same household cases. Of the 216 patients, 200 were tested for IgM & IgG antibodies (Abs) to Orthopoxviruses. All 200 patients had anti-orthopoxvirus IgG Abs; whereas 189/200 were positive for IgM. Patients with hypoalbuminemia had a high risk of severe disease. Patients with fatal disease had higher maximum geometric mean values than survivors for the following variables, respectively: viral DNA in blood (DNAemia); maximum lesion count; day of admission mean AST and ALT.

## Author summary

This is a prospective observational study of human mpox disease, an emerging infectious disease that has traditionally occurred in Central and West Africa. There are certain differential characteristics when compared to other pox diseases. We describe the presenting symptoms and signs of human mpox disease, laboratory findings and make recommendations for the medical treatment of patients with mpox disease. The classic MPX rash was seen in 99.5% of patients. Unlike other pox diseases, enlarged lymph nodes were observed in 98.6% of patients. The pathologic findings of MPXV infected placenta and fetus are presented as well.

## Introduction

Mpox virus, a zoonotic orthopoxvirus (OPXV), causes a potentially lethal infection in humans that clinically resembles smallpox [1]. Since the eradication of smallpox in the 1970’s [2], MPXV has been considered the OPXV posing the greatest danger to human populations [3]. Smallpox vaccination provides partial cross-protective immunity against human mpox [4–6]. The cessation of routine vaccination against smallpox has left human populations increasingly vulnerable to MPXV, and to variola virus (VARV, causative agent of smallpox), a potential agent of biowarfare [7].

Historically, most cases of mpox have occurred in western and central Africa, primarily the Democratic Republic of the Congo (DRC, formerly Zaïre) [3,8,9]. Intensive surveillance for human MPX from 1981 to 1986 [4] and stochastic modeling of MPXV transmission in human populations concluded that human to human transmission of mpox did not constitute a major public health problem [10]. However, more recent active surveillance undertaken in 2005–2007 demonstrated an average annual cumulative incidence of 5.53 per 10,000, a 20-fold increase over 30 years [11]. This finding has raised concerns over the potentially growing threat posed by MPXV and other OPXVs in the context of an increasingly immunologically naïve human population [12]. The re-emergence of human mpox infection, from 2017–2019, in West and Central Africa supports this proposition [13–16]. Furthermore, cases of mpox disease in humans have been imported into England, Singapore and Israel [17–19], in addition to the human cases in the United States stemming from rodents imported from Ghana [20] and a traveler from Nigeria to Dallas, TX [21]. More recent human-to-human transmission modeling of mpox by Grant, et al., concluded, “The geographic spread of mpox cases has expanded beyond the forests of central Africa, where cases were initially found, to other parts of the world, where cases have been imported. This transmission pattern is likely due to the worldwide decline in *orthopoxvirus* immunity, following cessation of smallpox vaccination.” They further used mathematical modeling to detail that “Monkeypox could therefore emerge as the most important *orthopoxvirus* infection in humans… [and] the epidemic potential of monkeypox will continue increasing” [22].

The intent of the study was to obtain human mpox infection data to include such parameters as lesion counts, levels of viremia and basic clinical lab tests to compare human mpox infection to various orthopox infection animal models of human smallpox. Since this study was initiated, treatments have been FDA approved for variola based on animal models; however, the continued use of such animal models will be important in exploration of additional therapeutic options for smallpox. The biodefense community recognizes a need for continued therapeutics development in the event of VARV reintroduction to the world as a human infection.

Since completion of this study, a world-wide outbreak of human mpox disease effecting over 80,000 people in over 100 countries has occurred. The currently circulating form of MPXD has been observed to effect the genital and anal regions effecting men who have sex with men more frequently than the general population and has an increased human-to-human transmission rate compared to classic MPXV infections [23,24,25].

A greater understanding of human MPXV infection will aid efforts to protect human populations from the threat posed by MPXV as well as the potential threat posed by VARV [26]. In this study, we sought to improve understanding of the clinical course of human MPXV infection which will be crucial for the continued development of therapeutic interventions against human OPXV infections.

## Methods

### Ethics statement

The study protocol was reviewed and approved by the Human Use Committee at the United States Army Medical Research Institute of Infectious Diseases (FY05-13) and the Headquarters, United States Army Medical Research and Development Command Institutional Review Board (IRB), Frederick, MD, USA, as well as the Ethics Committee at the University of Kinshasa School of Public Health (KSPH), Kinshasa, Democratic Republic of the Congo. The study was conducted according to the approved protocol and applicable U.S.A. federal, DOD and local regulatory requirements and guidelines as well as in compliance with applicable Congolese law. The study was conducted under the oversight of the Ministry of Health with appropriate guidance and collaboration from the KSPH and the INRB. All personnel involved in the study had human subjects protection training. Patient’s privacy was respected in keeping with local cultural and hospital standards. Reasonable care was taken to safeguard subject confidentiality and protect medical information consistent with limitations of the hospital facility. Informed consent/assent was obtained before any study procedure was performed. For children aged 0–11 years, written informed consent was obtained from parent or guardian. For minors 12–17 years, written assent was obtained as well as written parental/guardian informed consent. All were given a copy of their signed informed consent/assent. Informed consent document included permission for photographs to be taken by a member of the study team.

### Study site and population

The study site was Kole hospital in the Sankuru District of Kasaï-Oriental Province in DRC. Land cover in the Sankuru District consists of tropical rainforest, savannah, and traditional agricultural fields. Residents of the district are primarily subsistence farmers and hunters who live in small villages that average 100 individuals, spread amongst small clearings in the forest and small farming communes of extended families of less than 15 people [11]. The research was conducted jointly by the Institut National de Recherche Biomédicale (INRB) and the US Army Medical Research Institute of Infectious Diseases (USAMRIID) at one of the two previous WHO MPX study sites (1981–1986).

Admission to the “Mpox Isolation Ward” was based upon clinical diagnosis of human MPXV infection by hospital staff. A clinically overt case of active MPX was defined as having either (a) vesicular rash, with crops of vesicles of similar developmental stage in each body region (regional monomorphism) typically first appearing on the face, hands, and feet with centrifugal distribution (pox-like rash) or (b) fever of up to 38.5 to 40°C or a history of subjective fever, rash, lymphadenopathy, headache, malaise, or prostration in the past 2 weeks for which there is no attribution and have a history of one or more of the following exposures: handled or ate uncooked, freshly butchered meat, including meat of monkey, squirrel or other wild game during the 21 days prior to the onset of illness. These patients were informed about the observational study and given the option to participate by granting informed consent or if a minor, parental/legal guardian permission or ascent as the situation required.

Patients were typically accompanied by family members, who provided basic care for the patient. During this study family members stayed with the admitted patients within the isolation compound where they prepared food for patients and family members.

### Study design

Patients who presented to hospital admitting with a presumptive diagnosis of mpox were admitted to the physically separate infectious disease ward and offered an opportunity to be evaluated for enrollment in the study but received the same treatment independent of enrollment.

Once enrolled in this prospective observational study, continuation was based upon obtaining positive results with Pan-OPXV PCR. All Pan-OPXV PCR positive patients were also positive in the MPXV specific PCR. Positivity in any tissue was considered sufficient for inclusion in the PCR positive group.

Following the signing of the informed consent document, study personnel obtained a medical history; performed a physical examination; recorded vital signs and weight; conducted a complete lesion count; and collected blood for CBC, Chemistry, malaria smears; collected throat swabs, and urine. Other time points for CBC, viral culture, PCR were Days 1, 2, 3, 4, 5, 6, 8, 10,12, 14, day of discharge and day 75: clinical chemistry and U/A on Days 0, 4, 8, 12, day of discharge and day 75. Scabs were collected when available.

A pregnancy test was administered on day 0 to female participants of childbearing age but pregnant subjects were still allowed to enroll and participate. Onsite physicians examined patients on day of admission and the following 3 days, as well as the day of discharge and upon follow-up at day 75 (±15 days). The duration of hospitalization varied but was typically between 7 and 21 days. The severity of a given symptom or sign (mild, moderate, severe) upon admission or during hospital stay was not collected. We compared symptoms and physical findings using the CDC smallpox lesion count severity classification since there is no validated classification of lesions for human mpox disease.

### Adjunctive medications administered to patients under direction of hospital staff

In recognition that many patients were hospitalized with fever which may include other infectious processes in addition to or in lieu of mpox, patients were empirically treated with amoxicillin for the first few days until the etiology of the fever was clearly established. If the patients, had clinical suspicion of pneumonia or sepsis, the patients were treated with other available antibiotics. Because the Congo River basin has very high endemicity for Falciparum malaria, all patients with fever were treated empirically with appropriate antimalarial agents until such time as sequential malaria smears confirmed there was no detectable parasitemia or a full course of malaria treatment was completed if parasitemia was confirmed. A 10% solution of Potassium Permanganate was used to disinfect skin in conjunction with bathing and other hygienic measures to mitigate contact and fomite transmission of the infection.

Aspirin, acetaminophen and diclofenac were routinely given to reduce fever and treat pain, including painful adenopathy which patients frequently experienced. Mebendazole was given empirically on admission and continued for those who screened positive for evidence of helminth infection.

One of the most significant interventions that the hospital staff provided was nutritional support, which was given not only to the hospitalized patients, but also to those accompanying family members who provided primary nursing care, hygienic measures and emotional support to the hospitalized patients. When routine dietary intake was compromised by oropharyngeal lesions and painful cervical adenopathy attempt was made by the hospital staff to provide liquid food supplements and intravenous hydration.

### Lesion count methodology

An indelible marker was used by nurses to divide the patient’s body into nine different skin regions and the oropharynx when lesions involved the mouth and/or throat, for a total of 10 areas when the latter was involved. Total lesion count was determined by summing all types of lesions by study day. Results were audited by both the Clinical Monitor and study team quality control sections, utilizing the nurses’ case note—books that included lesion count by body area.

### Clinical laboratory procedures

Clinical laboratory studies were done in compliance with USAMRIID’s Clinical Laboratory procedures. Hematology was performed using an AcT10 Hematology Analyzer by Beckman Coulter, and urinalysis was done utilizing a Multistix 10 SG, by Siemens. Clinical chemistries were performed using a Piccolo Point of Care General Chemistry panel (ALB, ALP, ALT, AMY, AST, BUN, Ca, CRE, GGT, GLU, TBIL, TP) by Abaxis. HIV Ab were done at USAMRIID using the Clinical Laboratory’s routine HIV testing kit, Bio-Rad Laboratories Multispot HIV-1/HIV-2 Rapid Test. Throat swabs were done using routine daily clinical methodology.

### DNA extraction and PCR analysis methodology

DNA extraction with QIAamp DNA Mini Kit (Qiagen), inactivation of infectious virus by incubation of specimen in extraction buffer at 56° C for 1 hour, and pan-orthopox real time polymerase chain reaction (PCR) assays were performed using a Roche LightCycler and software [27]. All reagents were aliquoted into single test aliquots for up to 8 samples plus controls at USAMRIID. Each patient assay used new reagent aliquots done the day specimens were collected. Each assay included 6 standards covering the anticipated minimum and maximum values. Positive controls (EDTA blood spiked with gamma irradiated MPXV) and negative controls (EDTA blood only) were extracted with each assay run for specimens and results had to fall within expected values for extraction and assay to be valid. The PCR assay included samples to determine if contamination had occurred. In cases where controls did not meet requirements tests were repeated with new reagents and standards.

### IgM and IgG methodology

Serum IgM and IgG specific against anti-OPXV were detected by ELISA as previously described [28]. IgM and IgG assays were performed at 1:50 and 1:100 serum dilutions respectively using purified vaccinia virus DryVax strain. Samples were determined positive or negative based on the OD-COV value (Optical Density at 450 nm minus cutoff value of 3 times standard deviation of averages from 5 negative controls.

### Benefits to the Kole hospital and community

The study provided the following benefits to the Kole Hospital, staff and all patients: funding of salaries of hospital staff involved with care of mpox patients, supplementation of pharmaceutical and expendable supply budget, space available airlift of personnel and supplies, including food/nutritional support for mpox patients and their family caregivers, and funding of all medications provided to all MPX patients. The community had internet access with multiple terminals using 5% of the study internet satellite, and hospital physicians had unlimited access. Hospital clinicians had access to study clinical laboratory testing not routinely available at this hospital, as well as supplementation to the clinical staff with additional physicians to insure careful monitoring of disease and detection of treatable complications of illness.

A single payment of U.S. $20.00 was provided to study patients who returned for the follow up visit to compensate them for the blood draw. No other direct compensation was provided to study patients.

### Statistical methods

All statistical analyses were performed using SAS version 9.4 (SAS Institute, Cary, NC) and Statistical Package R version 3.6.1. P-values less than 0.05 (typically ≤ 0.05) were statistically significant [29]. Study data are available in 3 EXCEL files located in Supplemental Materials Section. Schematic drawings were created by study statisticians and the USAMRIID Visual Information Office.

Study Day 0 was defined as the admission day. Patients presented at various stages of disease on the admission day. Thus, for some analyses, the day of onset of rash was used to synchronize timing. Positive days were counted forward from Day 0 and negative days were counted backward from Day 0 after the rash onset. Patient age in years was computed as the integer value (nearest rounded year) based on the date of birth and the date of signed informed consent. Descriptive statistics were used to summarize the study results. PCR results and total lesion counts were log_10_ transformed for analyses. The age at admission was categorized as <5, 5–11, and ≥12 years. Repeated measurement Analysis of Variance (ANOVA) was used to compare means of total lesion count between different age groups. Repeated measurement ANOVA also was used to compare the means of blood PCR viral load between potential primary vs secondary cases or male vs female. Separate generalized estimating equations (GEE) with cumulative logit models were used to evaluate associations between: GI (anorexia, vomiting, abdominal pain, dysphagia, diarrhea, hepatomegaly, splenomegaly or both, abdominal tenderness) and HEENT (visual changes, eye pain/discharge, ear pain, nasal discharge/congestion, dysphagia, sore throat, conjunctive and other eye lesion, nasal discharge/congestion/ rhinorrhea/nasal lesion, mouth/throat lesions) clinical symptoms/signs severity (ordinal data) with total lesions, PCR results, clinical observations and vital signs; and total lesions severity (ordinal data) between vital signs, PCR results and clinical observations. Wilcoxon-Mann-Whitney test by ranks was utilized for comparison of continuous variables between two group and Kruskal Wallis Test for more than two groups. Fisher exact test or Chi-square test was used to assess binomial proportions between categorical variables. Cochran-Armitage test for trend was used to assess an increase or decrease in binomial proportions over ordered total lesion severity categories. The p values were adjusted by the stepdown Bonferroni correction method.

## Results

### Patient characteristics and demographics

Two-hundred forty-four (244) patients were enrolled in the study based upon the clinical diagnosis of MPXV infection. Of these, 216 patients had MPXV infection based upon Pan-Orthopox PCR positive and MPXV specific PCR positive results. The 216 patients with active MPXV infection were monitored and provided the data for this study. The first patient was enrolled 16 March 2007 and the last patient completed the study on 02 August 2011.

Patients were predominantly male (63.9%), age range 0–61 years (mean = 14, median = 13) (Table 1). Of the 216 patients, 118 (54.6%) were age ≥ 12. Four (4) patients reported history of smallpox vaccination, born during the years 1962–1972. However, 1 of 4 did not include the presence or absence of a scar notation. One patient was confirmed HIV positive based on Ab test; once recovered from acute MPXD, he was referred to a regional hospital for HIV treatment.

**Table 1:** Demographics and exposure history.

Characteristic	Age Group			Total
	< 5	5–11	≥ 12	
	(N = 31)	(N = 67)	(N = 118)	(N 216)
**Age at Admission (years), Mean (SD)**	2 (1.3)	8 (2.1)	21 (8.4)	14 (9.9)
**Gender, n (%)**				
Female	19 (61.3%)	20 (29.9%)	39 (33.1%)	78 (36.1%)
Male	12 (38.7%)	47 (70.1%)	79 (66.9%)	138 (63.9%)
**Marital Status, n (%)**				
Married	0 (0.0%)	0 (0.0%)	35 (29.7%)	35 (16.2%)
Single	31 (100.0%)	67 (100.0%)	83 (70.3%)	181 (83.8%)
**Family Exposure, n (%)** *				
Clean/Dressed Consumption of Wild Game	17 (54.8%)	53 (79.1%)	86 (72.9%)	156 (72.2%)
Handled Uncooked, Freshly Butchered Meat	14 (45.2%)	47 (70.1%)	72 (61.0%)	133 (61.6%)
Consumption of Ground Squirrel Meat	8 (25.8%)	30 (44.8%)	50 (42.4%)	88 (40.7%)
Initial Close Contact of Infected Individual (Household)	17 (54.8%)	23 (34.3%)	46 (39.0%)	86 (39.8%)
Consumption of Monkey Meat	9 (29.0%)	21 (31.3%)	52 (44.1%)	82 (38.0%)
Initial Contact with Blood, Body Fluids of Person with Illness Compatible with MPXD	12 (38.7%)	12 (17.9%)	33 (28.0%)	57 (26.4%)
Contact with Dead Animal (Not Killed by Self)	3 (9.7%)	17 (25.4%)	27 (22.9%)	47 (21.8%)
Other Wild Game Contact	8 (25.8%)	16 (23.9%)	22 (18.6%)	46 (21.3%)
Consumption of Gambian Rat or Other Rodent Meat	1 (3.2%)	5 (7.5%)	5 (4.2%)	11 (5.1%)
Multiple Exposures (≥ 2)	24 (77.4%)	67 (100.0%)	114 (96.6%)	205 (94.9%)
No Animal Exposure (Only Human Exposure)	10 (32.3%)	4 (6.0%)	14 (11.9%)	28 (13.0%)
No Human Exposure (Only Animal Exposure)	14 (45.2%)	44 (65.7%)	72 (61.0%)	130 (60.2%)
Both Human and Animal Exposure	7 (22.6%)	19 (28.4%)	32 (27.1%)	58 (26.9%)
Any Exposure	31 (100%)	67 (100%)	118 (100%)	216 (100%)

* “All meat was cooked before consumption”.

The overall mean length of hospital stay was 21.4 days; median = 22 days; minimum = 1; maximum 119 days. The mean, median, minimum-maximum days of stay based upon age group were: < 5 years = 24.6, 22.0, 2–119; 5 to <12 years = 21.2, 22.0, 1–73; ≥ 12 years = 20.7, 22, 4–92.

### Exposure history

All (100%) patients reported exposure to animal products or humans infected with MPXV. The most commonly reported exposure was cleaning/dressing/consumption of wild game (n = 156, 72.2%), followed by handling uncooked, freshly butchered meat (n = 133, 61.6%) and eating squirrel meat (n = 88, 40.7%) (Table 1). 205 (94.9%) patients had 2 or more possible exposures. Of the 11 individuals with a single exposure history, 4 were exposed to a household contact with MPXV infection, 4 consumed squirrel meat, 2 ate monkey meat and one cleaned, dressed, and consumed wild game (unspecified). All meat was cooked before consumption.

### Clinical symptoms and physical examination findings (signs)

The most common clinical complaint was rash (96.8%), followed by malaise (85.2%), sore throat (78.2%), lymphadenopathy (57.4%), anorexia (50.0%), cough (48.1%) and chills (44.5%) (Table 2). The clinical symptom occurred at least once from admission day (study day 0) to study day 3.

**Table 2:** Clinical Symptoms by age.

Organ-Systems	Clinical Symptom	Age Group						Total (N = 216)	
		<5 (N = 31)		5–11 (N = 67)		≥ 12 (N = 118)			
		During Study Days 0–3	Discharge	During Study Days 0–3	Discharge	During Study Days 0–3	Discharge	During Study Days 0–3	Discharge
		n (%)	n (%)	n (%)	n (%)	n (%)	n (%)	n (%)	n (%)
	Chills	18 (58.1)	1 (3.2)	28 (41.8)	1 (1.5)	51 (43.2)	0 (0.0)	97 (44.9)	2 (0.9)
General/Systemic	Fever	1 (3.2)	ND	0 (0.0)	ND	0 (0.0)	ND	1 (0.5)	ND
	Malaise	27 (87.1)	1 (3.2)	58 (86.6)	1 (1.5)	99 (83.9)	2 (1.7)	184 (85.2)	4 (1.9)
	Sweats	7 (22.6)	0 (0.0)	12 (17.9)	0 (0.0)	24 (20.3)	0 (0.0)	43 (19.9)	0 (0.0)
Skin/Derm	Rash	31 (100.0)	ND	66 (98.5)	ND	112 (94.9)	ND	209 (96.8)	ND
Lymphatics	Lymphadenopathy/Adenopathy	18 (58.1)	ND	40 (59.7)	ND	66 (55.9)	ND	124 (57.4)	ND
	Conjunctiva redness, Eye pain, Eye discharge, etc.	4 (12.9)	0 (0.0)	6 (9.0)	0 (0.0)	10 (8.5)	0 (0.0)	20 (9.3)	0 (0.0)
	Ear Pain	0 (0.0)	0 (0.0)	5 (7.5)	0 (0.0)	10 (8.5)	0 (0.0)	15 (6.9)	0 (0.0)
HEENT	Hard Of Hearing	0 (0.0)	ND	1 (1.5)	ND	0 (0.0)	ND	1 (0.5)	ND
	Mouth/Throat Lesions	5 (16.1)	ND	22 (32.8)	ND	26 (22.0)	ND	53 (24.5)	ND
	Nasal Discharge/Congestion	20 (64.5)	3 (9.7)	28 (41.8)	2 (3.0)	19 (16.1)	2 (1.7)	67 (31.0)	7 (3.2)
	Sore Throat	27 (87.1)	1 (3.2)	53 (79.1)	1 (1.5)	89 (75.4)	0 (0.0)	169 (78.2)	2 (0.9)
	Visual Changes	1 (3.2)	0 (0.0)	2 (3.0)	0 (0.0)	2 (1.7)	0 (0.0)	5 (2.3)	0 (0.0)
Cardiovascular	Chest Pain	0 (0.0)	0 (0.0)	1 (1.5)	0 (0.0)	10 (8.5)	4 (3.4)	11 (5.1)	4 (1.9)
	Swelling	1 (3.2)	ND	0 (0.0)	ND	0 (0.0)	ND	1 (0.5)	ND
Lungs	Cough	14 (45.2)	0 (0.0)	38 (56.7)	0 (0.0)	52 (44.1)	4 (3.4)	104 (48.1)	4 (1.9)
	Shortness of Breath	5 (16.1)	1 (3.2)	4 (6.0)	0 (0.0)	6 (5.1)	0 (0.0)	15 (6.9)	1 (0.5)
	Abdominal Pain	6 (19.4)	2 (6.5)	13 (19.4)	1 (1.5)	31 (26.3)	8 (6.8)	50 (23.1)	11 (5.1)
	Anorexia	20 (64.5)	1 (3.2)	34 (50.7)	1 (1.5)	54 (45.8)	0 (0.0)	108 (50.0)	2 (0.9)
Gastrointestinal	Diarrhea	3 (9.7)	1 (3.2)	2 (3.0)	1 (1.5)	5 (4.2)	1 (0.8)	10 (4.6)	3 (1.4)
	Dysphagia	6 (19.4)	0 (0.0)	22 (32.8)	0 (0.0)	26 (22.0)	0 (0.0)	54 (25.0)	0 (0.0)
	Vomiting	3 (9.7)	0 (0.0)	5 (7.5)	0 (0.0)	5 (4.2)	0 (0.0)	13 (6.0)	0 (0.0)
Hematologic	Bleeding, Active, Various Sites	0 (0.0)	0 (0.0)	0 (0.0)	0 (0.0)	6 (5.1)	0 (0.0)	6 (2.8)	0 (0.0)
	Petechiae	0 (0.0)	0 (0.0)	0 (0.0)	0 (0.0)	2 (1.7)	0 (0.0)	2 (0.9)	0 (0.0)
	Back Pain	0 (0.0)	0 (0.0)	2 (3.0)	0 (0.0)	23 (19.5)	0 (0.0)	25 (11.6)	0 (0.0)
	Cervical Deformation	0 (0.0)	ND	0 (0.0)	ND	1 (0.8)	ND	1 (0.5)	ND
	Joint Pain	0 (0.0)	0 (0.0)	5 (7.5)	0 (0.0)	15 (12.7)	1 (0.8)	20 (9.3)	1 (0.5)
Musculoskeletal	Muscle Pain	2 (6.5)	0 (0.0)	5 (7.5)	0 (0.0)	8 (6.8)	0 (0.0)	15 (6.9)	0 (0.0)
	Neck Stiffness	0 (0.0)	0 (0.0)	2 (3.0)	0 (0.0)	7 (5.9)	0 (0.0)	9 (4.2)	0 (0.0)
Neurologic	Dizziness	0 (0.0)	ND	0 (0.0)	ND	3 (2.5)	ND	3 (1.4)	ND
	Headache	0 (0.0)	0 (0.0)	16 (23.9)	1 (1.5)	35 (29.7)	0 (0.0)	51 (23.6)	1 (0.5)

During Study Days 0–3: The clinical symptom occurred at least once from the admission day (study day 0) to study day 3

The most common physical examination finding (clinical sign) was classic MPXV skin lesions 215/216 (99.5%) and lymphadenopathy (adenopathy) (98.6%) (Table 3). MPXV mouth/throat lesions were seen in 28.7% of patients. Hyperthermia was seen in 18.5%. Abnormal lung sounds were detectable in 10.6% of patients. Hepatomegaly, splenomegaly or both were noted in 7.9% of patients. Bleeding was seen in 2.3% of patients—bleeding sites included venipuncture sites, oral/gingival, rectal, nasal, conjunctival and vagina. Clinical signs were noted at least once from admission day (study day 0) to study day 3.

**Table 3:** Clinical signs (physical examination findings) by age.

Organ-Systems	Clinical Sign	Age Group						Total (N = 216)	
		<5 (N = 31)		5–11 (N = 67)		≥ 12 (N = 118)			
		During Study Days 0–3	Discharge	During Study Days 0–3	Discharge	During Study Days 0–3	Discharge	During Study Days 0–3	Discharge
		n (%)	n (%)	n (%)	n (%)	n (%)	n (%)	n (%)	n (%)
	Bed-bound	1 (3.2)	0 (0.0)	1 (1.5)	1 (1.5)	1 (0.8)	0 (0.0)	3 (1.4)	1 (0.5)
General/Systemic	Diminished Activity	26 (83.9)	0 (0.0)	49 (73.1)	0 (0.0)	74 (62.7)	0 (0.0)	149 (69.0)	0 (0.0)
	Hyperthermia	10 (32.3)	2 (6.5)	14 (20.9)	0 (0.0)	16 (13.6)	0 (0.0)	40 (18.5)	2 (0.9)
	Hypothermia	0 (0.0)	0 (0.0)	3 (4.5)	0 (0.0)	0 (0.0)	0 (0.0)	3 (1.4)	0 (0.0)
	Dehydration	0 (0.0)	0 (0.0)	3 (4.5)	0 (0.0)	4 (3.4)	0 (0.0)	7 (3.2)	0 (0.0)
Skin/Derm	Mpox Lesions	31 (100.0)	20 (64.5)	67 (100.0)	45 (67.2)	117 (99.2)	94 (79.7)	215 (99.5)	159 (73.6)
	Non-Specific Rash (Excludes MPX Legions)	23 (74.2)	15 (48.4)	45 (67.2)	26 (38.8)	70 (59.3)	58 (49.2)	138 (63.9)	99 (45.8)
Lymphatics	Lymphadenopathy/Adenopathy	31 (100.0)	26 (83.9)	67 (100.0)	57 (85.1)	115 (97.5)	91 (77.1)	213 (98.6)	174 (80.6)
	Conjunctival and Other Eye Lesion	2 (6.5)	2 (6.5)	6 (9.0)	0 (0.0)	6 (5.1)	0 (0.0)	14 (6.5)	2 (0.9)
HEENT	Mouth/Throat Lesions	7 (22.6)	0 (0.0)	25 (37.3)	0 (0.0)	30 (25.4)	0 (0.0)	62 (28.7)	0 (0.0)
	Nasal Discharge/Congestion/ Rhinorrhea/ Nasal Lesion	7 (22.6)	2 (6.5)	14 (20.9)	1 (1.5)	6 (5.1)	0 (0.0)	27 (12.5)	3 (1.4)
	Abnormal Heart Rhythms	0 (0.0)	0 (0.0)	2 (3.0)	0 (0.0)	3 (2.5)	0 (0.0)	5 (2.3)	0 (0.0)
Cardiovascular	Extremity Edema	1 (3.2)	0 (0.0)	0 (0.0)	0 (0.0)	5 (4.2)	0 (0.0)	6 (2.8)	0 (0.0)
	Heart Murmur	0 (0.0)	0 (0.0)	0 (0.0)	0 (0.0)	1 (0.8)	0 (0.0)	1 (0.5)	0 (0.0)
Lungs	Abnormal Lung Sounds	11 (35.5)	0 (0.0)	9 (13.4)	0 (0.0)	3 (2.5)	0 (0.0)	23 (10.6)	0 (0.0)
Gastrointestinal	Abdominal Tenderness	0 (0.0)	0 (0.0)	3 (4.5)	0 (0.0)	12 (10.2)	0 (0.0)	15 (6.9)	0 (0.0)
	Hepatomegaly, Splenomegaly or Both	2 (6.5)	0 (0.0)	5 (7.5)	0 (0.0)	10 (8.5)	1 (0.8)	17 (7.9)	1 (0.5)
Hematologic	Bleeding	0 (0.0)	0 (0.0)	0 (0.0)	0 (0.0)	5 (4.2)	0 (0.0)	5 (2.3)	0 (0.0)
Neurologic	Confused/Disoriented/ Lethargy/Stupor	3 (9.7)	0 (0.0)	4 (6.0)	1 (1.5)	6 (5.1)	0 (0.0)	13 (6.0)	1 (0.5)
Musculoskeletal	Musculoskeletal	0 (0.0)	1 (3.2)	1 (1.5)	0 (0.0)	4 (3.4)	1 (0.8)	5 (2.3)	2 (0.9)

During Study Days 0–3: The abnormal clinical sign occurred at least once from the admission day (study day 0) to study day 3. Hyperthermia defined by the highest temperature of patients on the admission day; Hypothermia defined by the first tested temperature of patients on the admission day.

**Fig 1:**
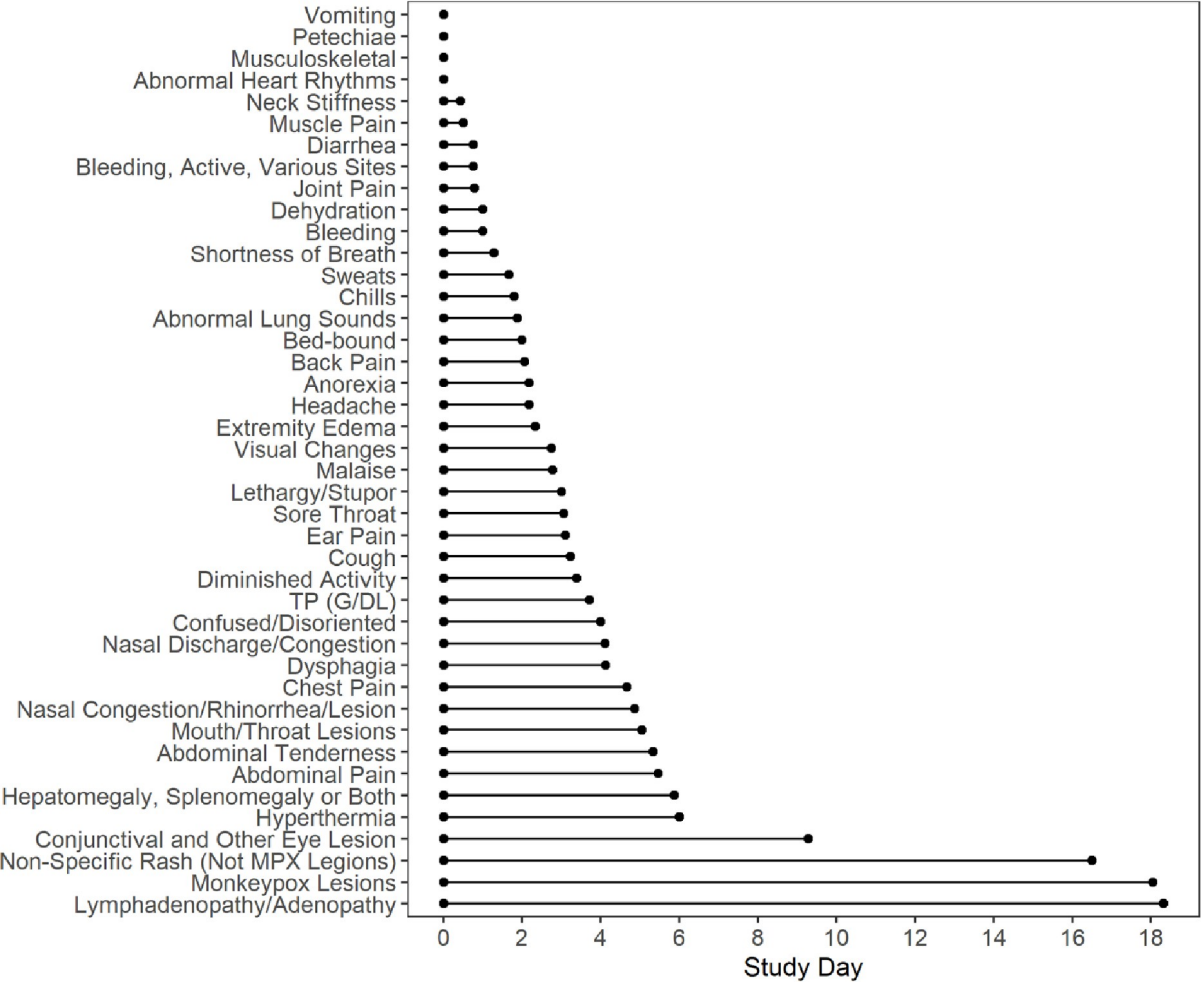
The mean of duration only included clinical symptoms and clinical signs present on the admission day.

Fig 1 shows the duration of symptoms and signs from admission day in patients with acute disease. As expected, at day of discharge the majority-of-patients still had evidence of mpox skin lesions and lymphadenopathy. However, most symptoms and signs lasted 3–5 days from admission.

### CDC lesion severity score and clinical symptoms & signs

Clinical symptoms and physical findings were compared across the CDC lesion severity categories (Tables 4 and 5). Both Fisher’s Exact Test/Chi-square Test and the Cochran-Armitage Trend Test showed significant adjusted P-values for rash, mouth/throat lesions, anorexia, and diminished activity (Fisher’s Exact Test range 0.0329 to <0.0001; Cochran-Armitage Trend Test p-value range 0.0084 to <0.0001). The Cochran-Armitage Trend Test also showed significant adjusted P-value for nasal discharge/congestion (p-value = 0.0084).

**Table 4:** Comparison of clinical symptoms across lesion severity categories.

		Total Lesion Severity Score							
Organ-Systems	Clinical Symptom	<25	25–99	100–499	> = 500	Fisher’s Exact Test/ Chi-square Test		Cochran-Armitage Trend Test	
		(N = 20)	(N = 58)	(N = 91)	(N = 47)				
		n (%)	n (%)	n (%)	n (%)	Adjusted P value	Raw P value	Adjusted P value	Raw P value
	Chills	10 (50.0)	17 (29.3)	41 (45.1)	29 (61.7)	0.2160	0.0098	0.4648	0.0227
General	Malaise	18 (90.0)	47 (81.0)	73 (80.2)	46 (97.9)	0.2846	0.0136	1.0000	0.1678
	Sweats	0 (0.0)	7 (12.1)	24 (26.4)	12 (25.5)	0.1821	0.0079	0.0899	0.0039
Skin/Derm	Rash	16 (80.0)	56 (96.6)	90 (98.9)	47 (100.0)	**0.0329**	0.0013	**0.0084**	0.0003
Lymphatics	Lymphadenopathy/Adenopathy	13 (65.0)	35 (60.3)	43 (47.3)	33 (70.2)	1.0000	0.0540	1.0000	0.8442
	Conjunctiva redness, Eye pain, Eye discharge, etc.	0 (0.0)	5 (8.6)	6 (6.6)	9 (19.1)	1.0000	0.0559	0.4648	0.0221
	Ear Pain	0 (0.0)	4 (6.9)	6 (6.6)	5 (10.6)	1.0000	0.5886	1.0000	0.1743
HEENT	Mouth/Throat Lesions	1 (5.0)	8 (13.8)	24 (26.4)	20 (42.6)	**0.0265**	0.0010	**0.0019**	< .0001
	Nasal Discharge/Congestion	3 (15.0)	11 (19.0)	30 (33.0)	23 (48.9)	0.0952	0.0040	**0.0084**	0.0003
	Sore Throat	15 (75.0)	42 (72.4)	71 (78.0)	41 (87.2)	1.0000	0.2968	1.0000	0.1009
	Visual Changes	0 (0.0)	1 (1.7)	3 (3.3)	1 (2.1)	1.0000	1.0000	1.0000	0.5505
Cardiovascular	Chest Pain	1 (5.0)	3 (5.2)	5 (5.5)	2 (4.3)	1.0000	1.0000	1.0000	0.8892
Lungs	Cough	8 (40.0)	24 (41.4)	43 (47.3)	29 (61.7)	1.0000	0.1676	0.6651	0.0391
	Shortness of Breath	1 (5.0)	1 (1.7)	6 (6.6)	7 (14.9)	1.0000	0.0719	0.4648	0.0241
	Abdominal Pain	6 (30.0)	11 (19.0)	21 (23.1)	12 (25.5)	1.0000	0.7069	1.0000	0.8845
	Anorexia	7 (35.0)	22 (37.9)	44 (48.4)	35 (74.5)	**0.0199**	0.0007	**0.0051**	0.0002
Gastrointestinal	Diarrhea	0 (0.0)	1 (1.7)	7 (7.7)	2 (4.3)	1.0000	0.3476	1.0000	0.2239
	Dysphagia	2 (10.0)	11 (19.0)	25 (27.5)	16 (34.0)	1.0000	0.1235	0.3466	0.0158
	Vomiting	0 (0.0)	1 (1.7)	7 (7.7)	5 (10.6)	1.0000	0.1553	0.4648	0.0238
Hematologic	Bleeding, Active, Various Sites	0 (0.0)	1 (1.7)	3 (3.3)	2 (4.3)	1.0000	0.9282	1.0000	0.2635
	Petechiae	0 (0.0)	0 (0.0)	1 (1.1)	1 (2.1)	1.0000	0.7727	1.0000	0.2425
	Back Pain	3 (15.0)	4 (6.9)	12 (13.2)	6 (12.8)	1.0000	0.5600	1.0000	0.6511
	Joint Pain	3 (15.0)	2 (3.4)	7 (7.7)	8 (17.0)	1.0000	0.0648	1.0000	0.2154
Musculoskeletal	Muscle Pain	0 (0.0)	6 (10.3)	5 (5.5)	4 (8.5)	1.0000	0.4428	1.0000	0.6447
	Neck Stiffness	1 (5.0)	0 (0.0)	5 (5.5)	3 (6.4)	1.0000	0.1961	1.0000	0.2344
Neurologic	Dizziness	0 (0.0)	0 (0.0)	3 (3.3)	0 (0.0)	1.0000	0.4998	1.0000	0.6454
	Headache	4 (20.0)	13 (22.4)	22 (24.2)	12 (25.5)	1.0000	0.9705	1.0000	0.5860

Total lesion severity score is total number of lesions present on admission day. Differences between categories tested by Fisher exact test or Chi-square test with stepdown Bonferroni correction. Trend (decreasing or increasing) across categories tested by Cochran-Armitage Trend Test with stepdown Bonferroni correction. Significant differences were **bolded** (adjusted *p* value ≤ 0.05)

**Table 5:** Comparison of clinical signs between lesion severity score.

Organ-Systems	Clinical Sign	Total Lesion Severity Score				Fisher’s Exact Test/ Chi-square Test		Cochran-Armitage Trend Test	
		<25	25–99	100–499	> = 500				
		(N = 20)	(N = 58)	(N = 91)	(N = 47)				
		n (%)	n (%)	n (%)	n (%)	Adjusted P value	Raw P value	Adjusted P value	Raw P value
	Bed-bound	0 (0.0)	2 (3.4)	0 (0.0)	1 (2.1)	1.0000	0.2023	1.0000	0.8497
General/Systemic	Diminished Activity	6 (30.0)	31 (53.4)	69 (75.8)	43 (91.5)	**< .0001**	< .0001	**< .0001**	< .0001
	Hyperthermia	4 (20.0)	7 (12.1)	17 (18.7)	12 (25.5)	1.0000	0.3498	1.0000	0.2071
	Hypothermia	0 (0.0)	1 (1.7)	1 (1.1)	1 (2.1)	1.0000	1.0000	1.0000	0.6454
	Dehydration	0 (0.0)	1 (1.7)	3 (3.3)	3 (6.4)	1.0000	0.5479	1.0000	0.1168
Skin/Derm	Non-Specific Rash (Excludes MPX Legions)	15 (75.0)	42 (72.4)	52 (57.1)	29 (61.7)	1.0000	0.1946	1.0000	0.0991
Lymphatics	Lymphadenopathy/Adenopathy	19 (95.0)	57 (98.3)	90 (98.9)	47 (100.0)	1.0000	0.4265	1.0000	0.1365
	Conjunctival and Other Eye Lesion	0 (0.0)	3 (5.2)	5 (5.5)	6 (12.8)	1.0000	0.2534	0.7207	0.0515
HEENT	Mouth/Throat Lesions	3 (15.0)	11 (19.0)	29 (31.9)	19 (40.4)	0.7650	0.0450	0.0929	0.0052
	Nasal Discharge/Congestion/Rhinorrhea/Nasal Lesion	1 (5.0)	3 (5.2)	13 (14.3)	10 (21.3)	0.9400	0.0588	0.1516	0.0089
Cardiovascular	Abnormal Heart Rhythms	0 (0.0)	0 (0.0)	2 (2.2)	3 (6.4)	1.0000	0.2023	0.5178	0.0345
	Extremity Edema	0 (0.0)	0 (0.0)	5 (5.5)	1 (2.1)	1.0000	0.2436	1.0000	0.2635
Lungs	Abnormal Lung Sounds	0 (0.0)	6 (10.3)	9 (9.9)	8 (17.0)	1.0000	0.2263	0.8709	0.0670
Gastrointestinal	Abdominal Tenderness	1 (5.0)	6 (10.3)	8 (8.8)	2 (4.3)	1.0000	0.7207	1.0000	0.5749
	Hepatomegaly, Splenomegaly or Both	0 (0.0)	1 (1.7)	3 (3.3)	1 (2.1)	1.0000	1.0000	1.0000	0.5505
Hematologic	Bleeding	1 (5.0)	0 (0.0)	3 (3.3)	1 (2.1)	1.0000	0.3099	1.0000	0.9273
Neurologic	Confused/Disoriented/Lethargy/Stupor	1 (5.0)	0 (0.0)	5 (5.5)	7 (14.9)	0.2201	0.0116	0.1680	0.0099
Musculoskeletal	Musculoskeletal	2 (10.0)	4 (6.9)	6 (6.6)	3 (6.4)	1.0000	0.9172	1.0000	0.6627

Total lesion severity score equals total number of lesions present on admission day. Differences between categories tested by Fisher exact test or Chi-square test with stepdown Bonferroni correction. Trend (decreasing or increasing) across categories tested by Cochran-Armitage Trend Test with stepdown Bonferroni correction. Significant differences were **bolded** (adjusted *p* value ≤ 0.05).

### Mpox rash/lesion distribution and characteristics

**Fig 2:**
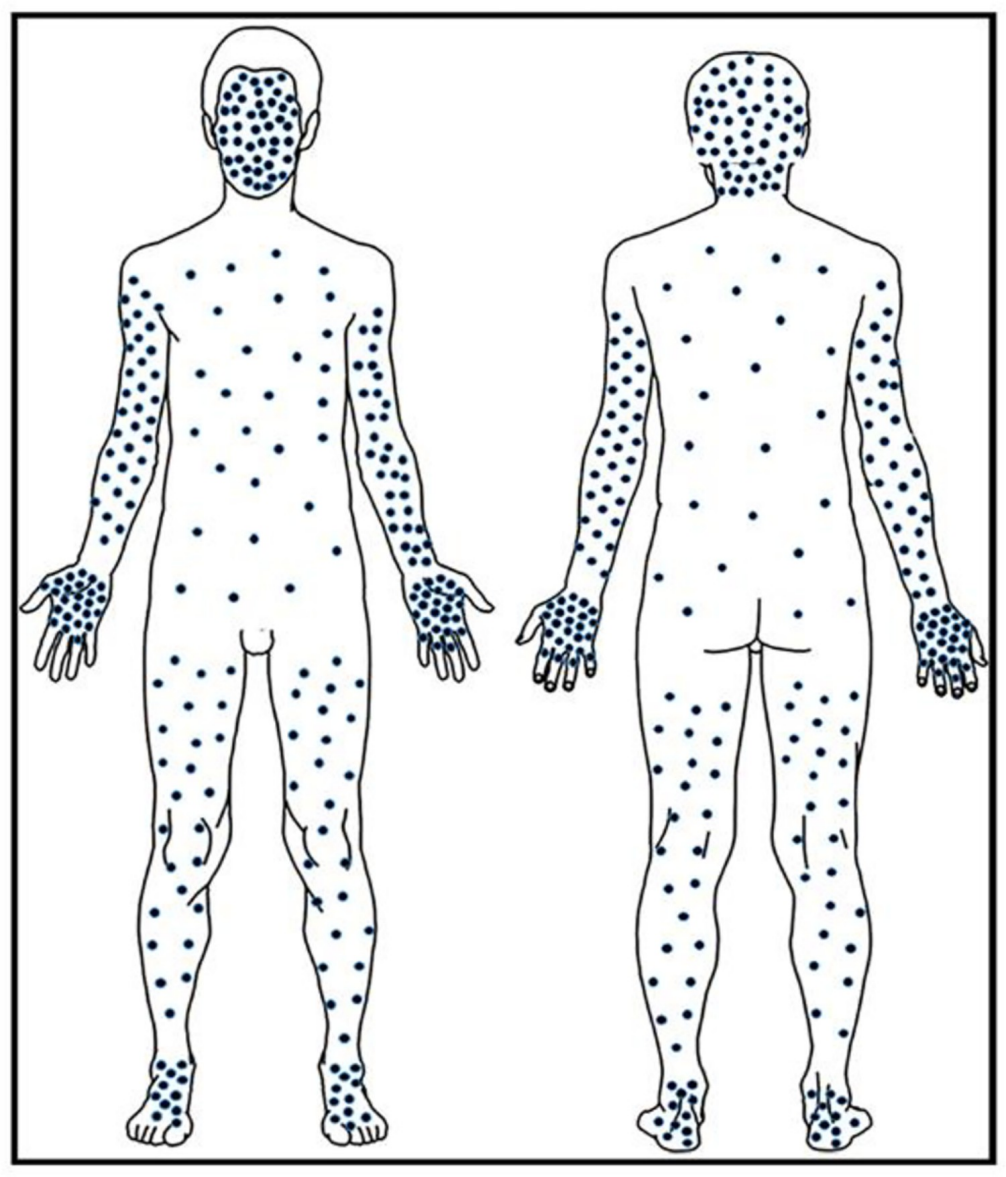
The figure provides a graphical representation of relative lesion density for dermatological regions. Each dot notionally represents approximately 10 lesions per body region as a function of the region’s relative surface area (mean of lesion count/percentage of surface area).

**Fig 3:**
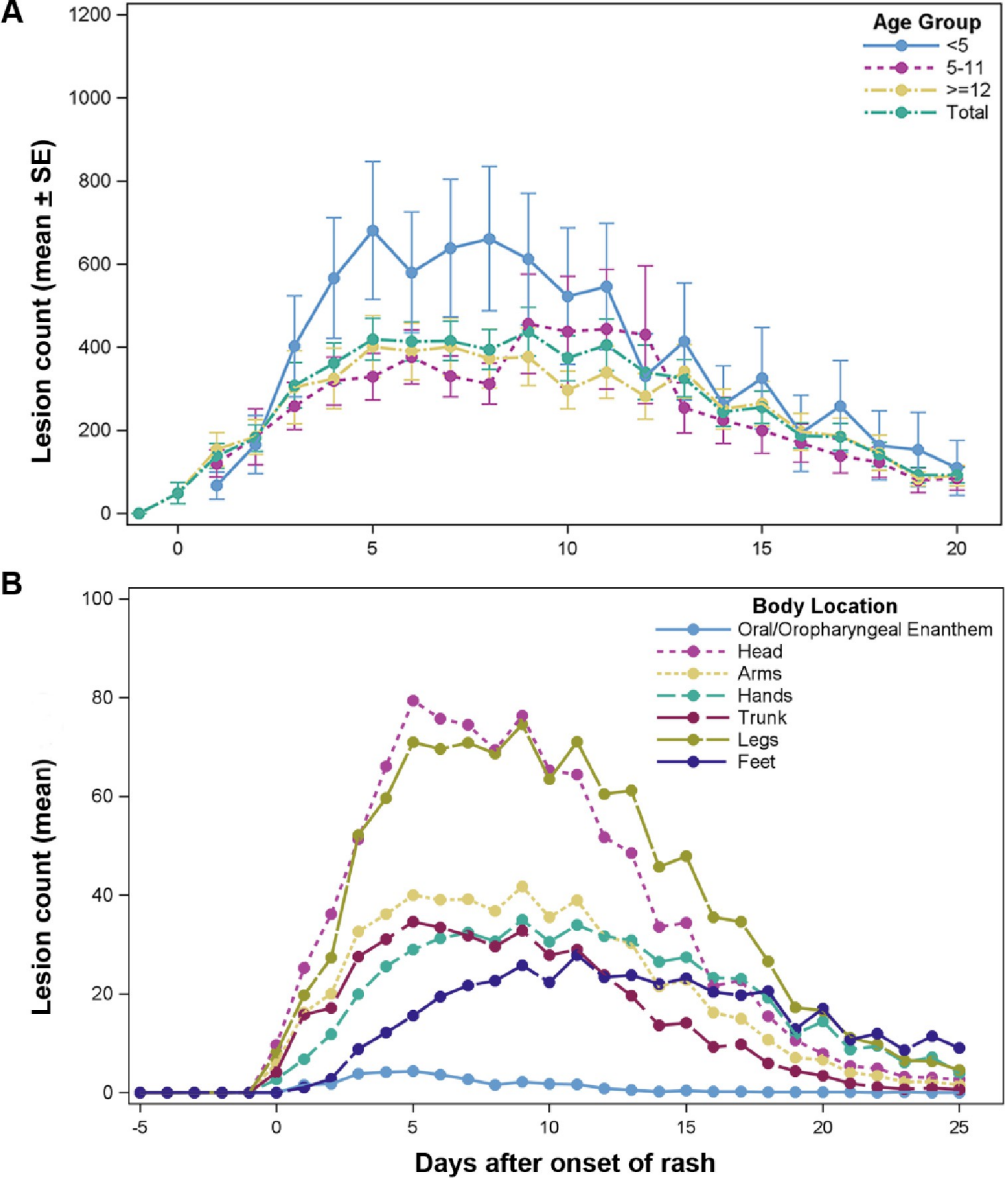
The mean with standard error bars of total lesion count by age group. (A) Repeated measurement Analysis of Variance (ANOVA) showed there was no significant difference among the groups (*p* = 0.2552). (B) Distribution of mean of lesion counts for all patients by body location over time.

Generally, mpox lesions were concentrated away from the trunk in a centrifugal pattern on the head/face, arms/hand and legs/feet (Fig 2) as shown in this diagram of the mean lesion count for patients in this study. Fig 3A shows the mean (±SE) total lesion count by age group over time from day of onset. The mean lesion count was higher for the age group <5 years compared to age groups 5–11 and ≥12 years, although the difference was not statistically significant (p = 0.1630). Lesions peaked between days 5–8.

Lesion counts by body region over time is shown in Fig 3B. Lesion counts are highest and peaked first on the head/face and extremities. Patient lesion distribution by age group and CDC lesion severity score is shown in Table 6.

**Table 6:** Descriptive table of lesion severity score by age.

Total Lesion Severity Score	Age Group			Total
	< 5	5–11	≥ 12	
	(N = 31)	(N = 67)	(N = 118)	(N 216)
	n (%)	n (%)	n (%)	n (%)
<25	0 (0)	6 (9.0)	14 (11.9)	20 (9.3)
25–99	5 (16.1)	18 (26.9)	35 (29.7)	58 (26.9)
100–499	15 (48.4)	27 (40.3)	49 (41.5)	91 (42.1)
> = 500	11 (35.5)	16 (23.9)	20 (17.0)	47 (21.8)

Total lesion severity score equals total number of lesions present on admission day.

**Fig 4:**
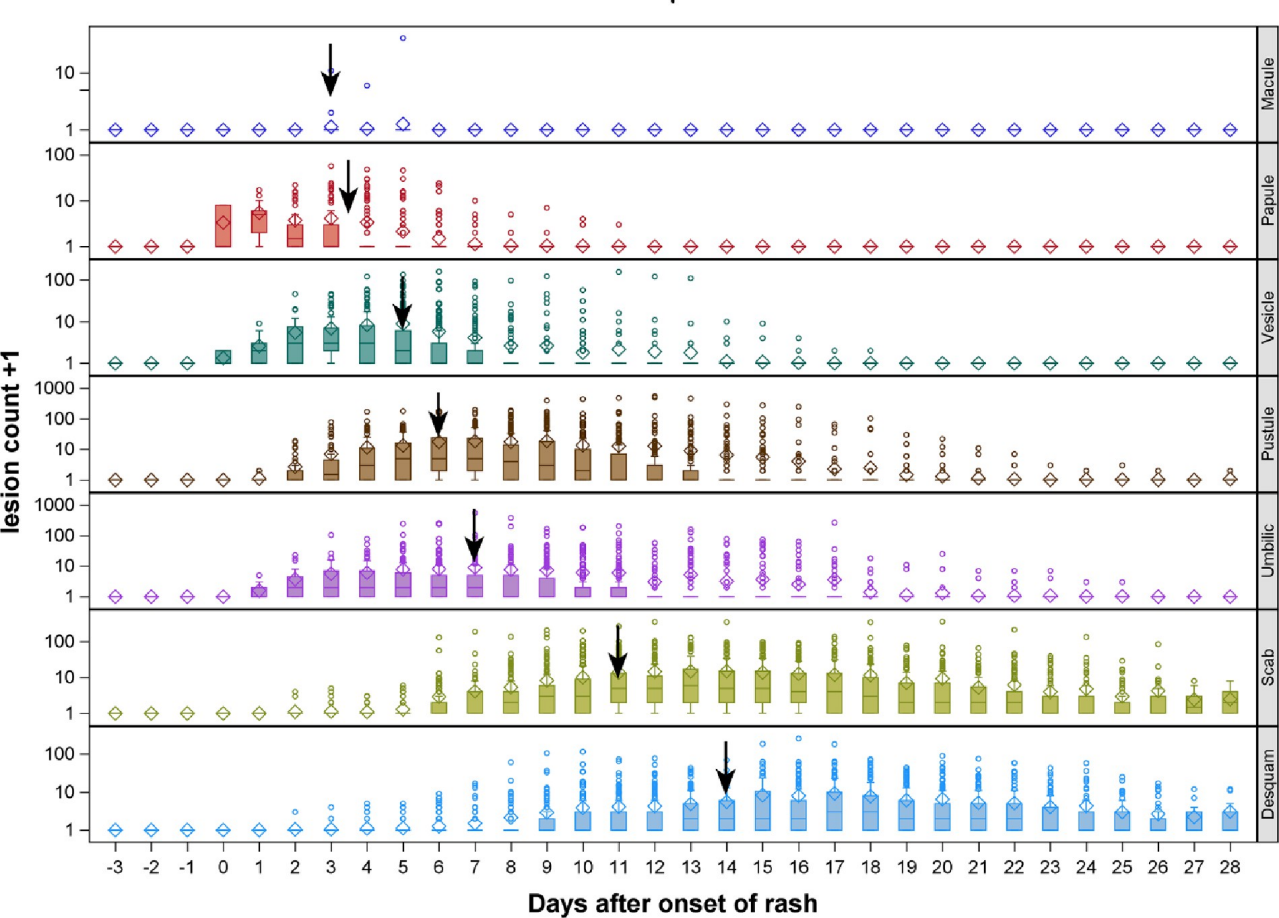
Lesion progression from macule, papule, vesicle, pustule, umbilication (Umbilic), scabbing (scab) and desquamation (desquam). **◊** represent mean of count, **─** represent median of count, ° represent outliers, **↓** represent median day after onset of rash to get max lesion count for that specific rash morphology on hand.

Fig 4 shows lesion progression from macule, papule, vesicle, pustule, umbilication, scabbing and desquamation for the hand. Progression from one phase to another occurs in order previously documented. Classic dogma holds that lesions in the same body region progress together as illustrated here.

### Mpox infection associated lymphadenopathy

**Fig 5:**
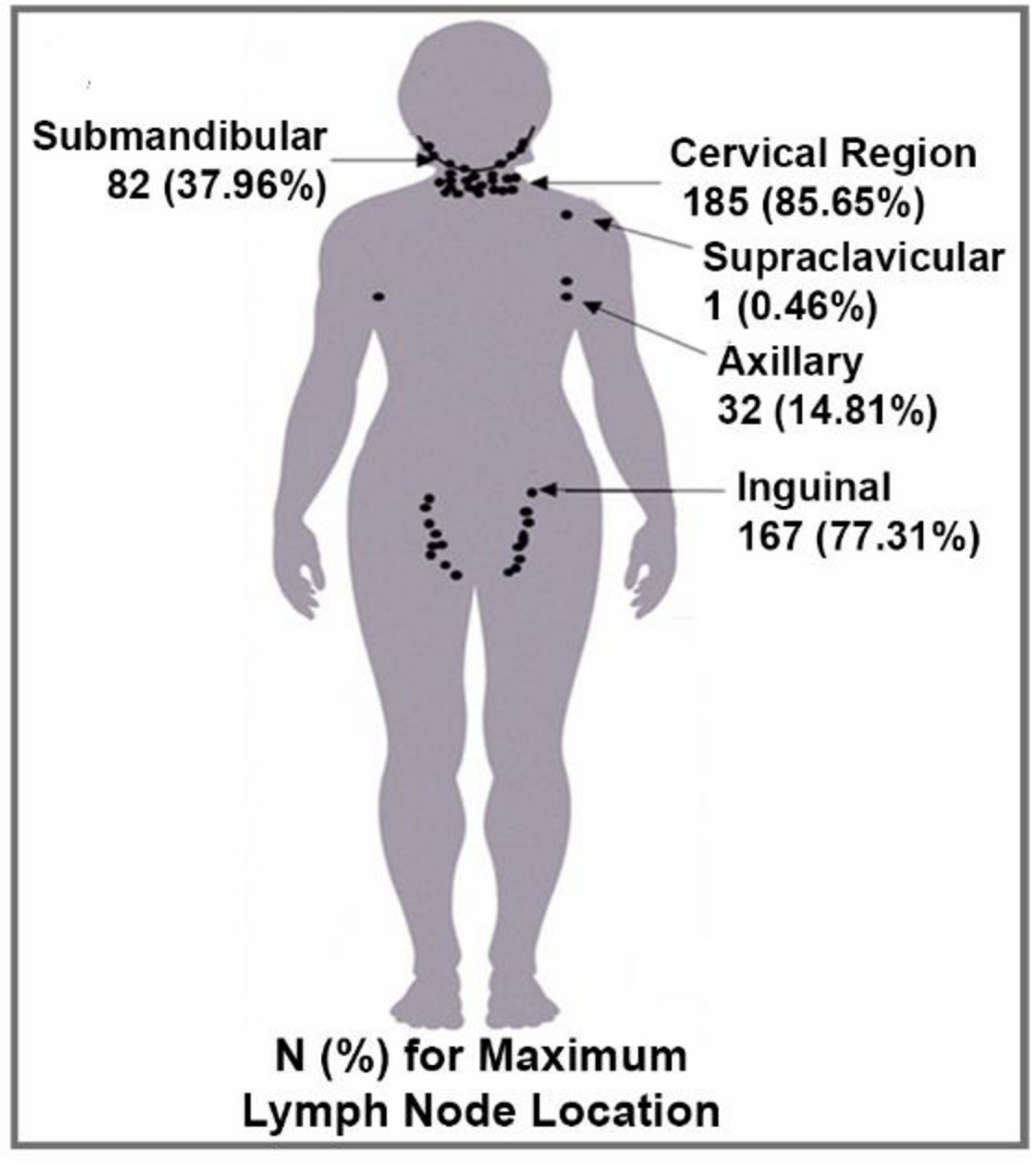
Mpox associated lymph node distribution. The lymph node count and distribution were documented on the day patients had the most lymph nodes. One dot equals 10 lymph nodes or fraction thereof depending upon the count.

The frequency of mpox induced lymphoadenopathy was 98.6% (Table 3), second only to the frequency of the classic mpox rash itself. The distribution of lymphoadenopathy in this study is depicted in Fig 5. The cervical region was most frequently afflicted at 85.6%; the second most frequent area was the inguinal region 77.3%.

### Clinical laboratory findings

Total white blood cell count, neutrophil and lymphocyte counts are compared to lesion severity count in Table 7. The median value for each increase as the lesion count increases.

**Table 7:** Comparison of CBC values between lesion severity score.

	Total Lesion Severity Score									
Test	<25 (N = 20)		25–99 (N = 58)		100–499 (N = 91)		> = 500 (N = 47)		Adjusted P value	Raw P value
	n	Median (Min-Max)	n	Median (Min-Max)	n	Median (Min-Max)	n	Median (Min-Max)		
Hg (gm/dL)	17	11.20 (7.9–15.9)	44	11.50 (7.1–16.5)	66	11.45 (7.1–14.7)	34	11.20 (8.4–16.4)	1.0000	0.7150
HCT (%)	20	33.10 (24.0–45.4)	58	34.05 (24.0–49.8)	91	34.30 (23.6–47.5)	47	34.00 (24.9–54.0)	1.0000	0.8746
WBC (10^3 cell/)	19	6.60 (3.2–12.4)	56	8.35 (5.0–27.5)	91	9.20 (3.5–40.1)	47	12.10 (6.3–42.8)	**< .0001**	< .0001
Neut (cell/mm^3)	19	2025.0 (400–5208)	56	2694.0 (715–8517)	91	3744.0 (896–13233)	46	5018.0 (1744–18270)	**< .0001**	< .0001
Lymph (cell/mm^3)	19	2832.0 (992–8085)	56	3750.0 (0–17325)	91	4224.0 (872–20852)	46	4568.0 (1634–19184)	**0.0349**	0.0070
EOSIN (cell/mm^3)	19	767.0 (312–2025)	56	1024.0 (0–6732)	91	891.0 (0–6023)	46	1180.0 (0–5550)	1.0000	0.8199
PLT (10^3/UL)	19	194.0 (75–661)	56	240.0 (27–668)	91	260.0 (89–1075)	47	246.0 (1–1120)	0.7547	0.1887

CBC tests by total lesion severity score on admission day. Statistics performed by Kruskal Wallis Test with stepdown Bonferroni correction.

Median clinical laboratory hepatic and renal functional tests did not show a relationship to lesion counts (Table 8).

**Table 8:** Comparison of clinical laboratory values and lesion severity score.

	Total Lesion Severity Score									
Test	<25 (N = 20)		25–99 (N = 58)		100–499 (N = 91)		> = 500 (N = 47)		Adjusted P value	Raw P value
	n	Median (Min-Max)	n	Median (Min-Max)	n	Median (Min-Max)	n	Median (Min-Max)		
ALB (G/DL)	20	2.90 (2.1–7.7)	54	2.80 (1.3–3.3)	86	2.70 (1.5–3.7)	46	2.60 (1.9–3.5)	0.1366	0.0137
ALP (U/L)	20	107.0 (1–238)	54	115.5 (47–242)	84	114.5 (57–238)	44	107.5 (48–304)	1.0000	0.3541
ALT (U/L)	20	24.0 (3–43)	54	21.0 (7–59)	86	24.0 (6–174)	46	21.0 (3–201)	1.0000	0.4744
AMY (U/L)	20	72.0 (25–200)	54	65.0 (8–161)	86	65.0 (6–1061)	45	43.0 (3–215)	0.1123	0.0102
AST (U/L)	20	38.0 (23–87)	54	36.5 (9–171)	86	39.5 (13–282)	45	44.0 (18–865)	0.3047	0.0339
BUN (MG/DL)	19	8.0 (1–14)	50	6.0 (1–13)	75	7.0 (1–19)	43	9.0 (2–32)	0.0806	0.0067
CA (MG/DL)	20	8.80 (2.7–9.3)	54	8.90 (4.2–10.1)	86	9.00 (7.4–9.8)	45	8.90 (7.5–10.2)	1.0000	0.1794
CRE (MG/DL)	20	0.70 (0.4–5.5)	54	0.65 (0.3–1.3)	84	0.60 (0.1–1.4)	44	0.60 (0.1–1.4)	1.0000	0.3254
GGT (U/L)	20	32.0 (15–157)	54	27.0 (3–197)	86	30.5 (7–703)	46	30.0 (11–391)	1.0000	0.6695
GLU (MG/DL)	20	89.5 (63–113)	54	89.5 (53–127)	86	95.0 (41–170)	46	96.5 (60–209)	0.6767	0.0967
TBIL (MG/DL)	20	0.70 (0.5–1.3)	54	0.50 (0.3–1.3)	86	0.60 (0.3–1.2)	45	0.60 (0.3–2.4)	0.3539	0.0442
TP (G/DL)	20	7.60 (6.9–13.0)	54	7.90 (3.2–9.5)	86	7.80 (5.4–9.6)	46	7.65 (6.1–10.1)	1.0000	0.9009

Clinical laboratory tests by total lesion severity score on admission day. Statistics performed by Kruskal Wallis Test with stepdown Bonferroni correction.

CBC, clinical laboratory and urinary protein severity classification (mild, moderate, severe, potentially life threatening) are presented by age and total lesion score in S1–S3 and S4–S6 Tables, respectively.

### IgM and IgG antibody responses

A total of 173 patients serum samples were tested for IgM and IgG by ELISA. A total of 189 (94.5%) develop IgM responses and all 173 (100%) were either IgG positive at enrollment or became positive during their hospitalization. The results of GEE with a cumulative logit model showed that total lesion severity was not significantly associated with IgG antibody responses (OR = 1.38, 95% CI: 0.72–2.62, p = 0.3597). However, the same model showed IgM antibody responders were 5.09 times more likely to have higher lesion severity association than IgM non-responders (OR = 5.09, 95% CI: 2.91–8.93, p < 0.0001).

### PCR results

**Fig 6:**
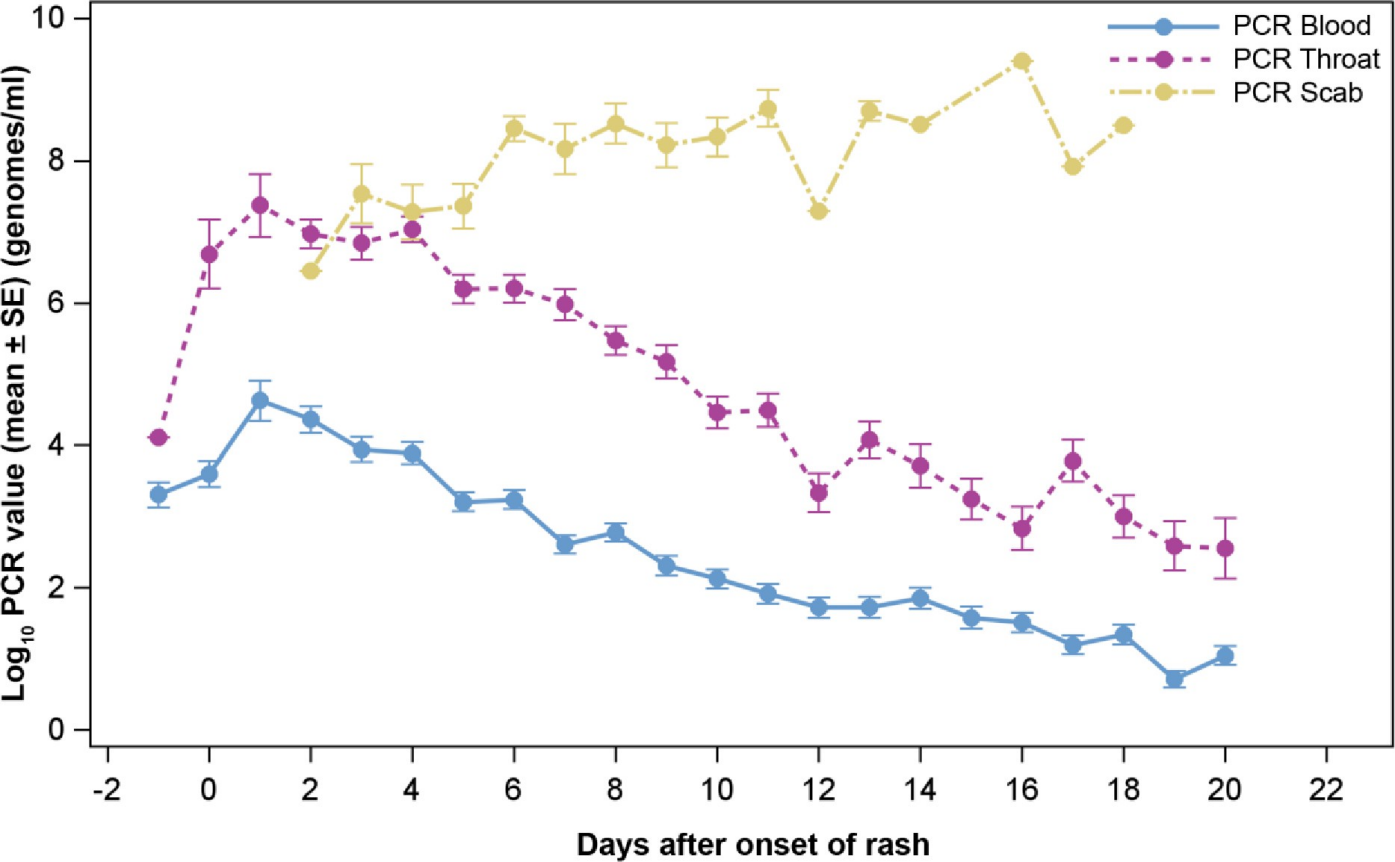
Mean of PCR count from blood, throat and scab (Log_10_) with standard error bars (Unit: genomes/mL) over time.

Fig 6 shows MPXV genomic DNA detection in blood and pharyngeal swabs by PCR occurs before the onset of the classic rash onset. Therefore, care should be taken when comparing PCR results with other variables because the maximum PCR viral load in blood occurs near the first day of rash appearance, often before patients present to the hospital. Unlike maximum lesion count, maximum blood PCR viral load often could not be accurately determined in this study—by the time most patients arrived at the hospital, viral load would have already peaked. The graphs show a decrease in viral load over time for blood (p < 0.0001) and pharynx (p < 0.0001). Generally, for specimens collected at the same time from the same patient, the PCR viral load from throat is about 2000 genomes/mL higher than blood.

There was no difference in the peak level and decline over time for males and females for blood (p = 0.9079) or pharynx (p = 0.5208) PCR copy numbers. The Wilcoxon-Mann-Whitney test showed no significant differences in maximum PCR blood or maximum PCR throat swabs results (p = 0.4505 and 0.8778, respectively). Scabs contain significant quantities of MPXV positive DNA until and including when they fall off. The concentration of MPXV in the scab is several times the number of genomes in blood and throat. Viral infectivity in specimens was not determined.

A strong association is seen between lesion severity scores and mean PCR DNA values from blood, throat and scab (Table 9).

**Table 9:** Descriptive table of admission day PCR values by lesion severity score.

	Total Lesion Severity Score								
PCR (Log_10_ genomes/ml)	<25		25–99		100–499		> = 500		Adj. p value
	(N = 20)		(N = 58)		(N = 91)		(N = 47)		
	n	Mean (SE)	n	Mean (SE)	n	Mean (SE)	n	Mean (SE)	
PCR Blood	19	2.9 (0.40)	56	2.7 (0.27)	90	3.7 (0.19)	45	4.4 (0.31)	**< .0001**
PCR Throat	6	4.3 (0.91)	21	5.8 (0.55)	47	6.5 (0.29)	24	7.4 (0.34)	**< .0001**
PCR Scab	1	6.6 (.)	2	6.2 (2.67)	12	7.7 (0.72)	8	8.6 (0.31)	**0.0123**

Log10 PCR values were compared using separate generalized estimating equations (GEE) with cumulative logit models, day after rash onset was adjusted as covariate. Adjusted *p* values were calculated using stepdown Bonferroni correction if needed. Total lesion severity score equals total number of lesions present on admission day.

### Secondary household cases of Mpox

For this analysis, patients who developed skin rash 14 days or longer after the first household case of mpox were labeled secondary cases. Of the 216 patients in the study, 105 had family relationships. Twelve of the 44 families (27.27%) or 18 family members in this study had secondary infections by this definition. The rate of secondary infection compared to the number of patients with family relationships was 17.4% (18/105). Patients diagnosed as secondary cases had a lower mean total lesion count (386 vs 618) that peaked at day 4–5 vs. 9–10 days and healed at a faster rate than primary cases. Classic primary and secondary cases are presented below.

- A professional hunter presented to the hospital with primary mpox disease with a fever of 7 days and pox lesions present for 5 days with an admission lesion count of 427 that peaked on lesion day 10 with 751 lesions. On admission the patient had severe lymphadenopathy with blood MPX PCR viral load 2.1 X 10^5^ genomes/ml, the maximum value seen in this patient, that slowly decreased to 1.5 X 10^3^ by lesion day (LD) 19. The patient was discharged after 34 days of hospitalization, much longer than normal.

A household member, presented with secondary mpox with enlarged cervical lymph nodes (10 mm), positive blood mpox PCR viral load (4.8 10^3^ genomes/mL) and throat swab (1.4 X 10^6^ genomes). Lesions were not present on admission and first appeared on the 3^rd^ hospital day (HD) LD 0 with 3 lesions that reached a maximum on HD 6 (LD 4) with 21 lesions and blood genomes reached a maximum on HD 5 with 1.3 X 10^5^ genomes/ml that slowly decreased to 3.9 X 10^2^ on discharge, HD 18.

### Pregnancies and fetal outcomes

**Fig 7:**
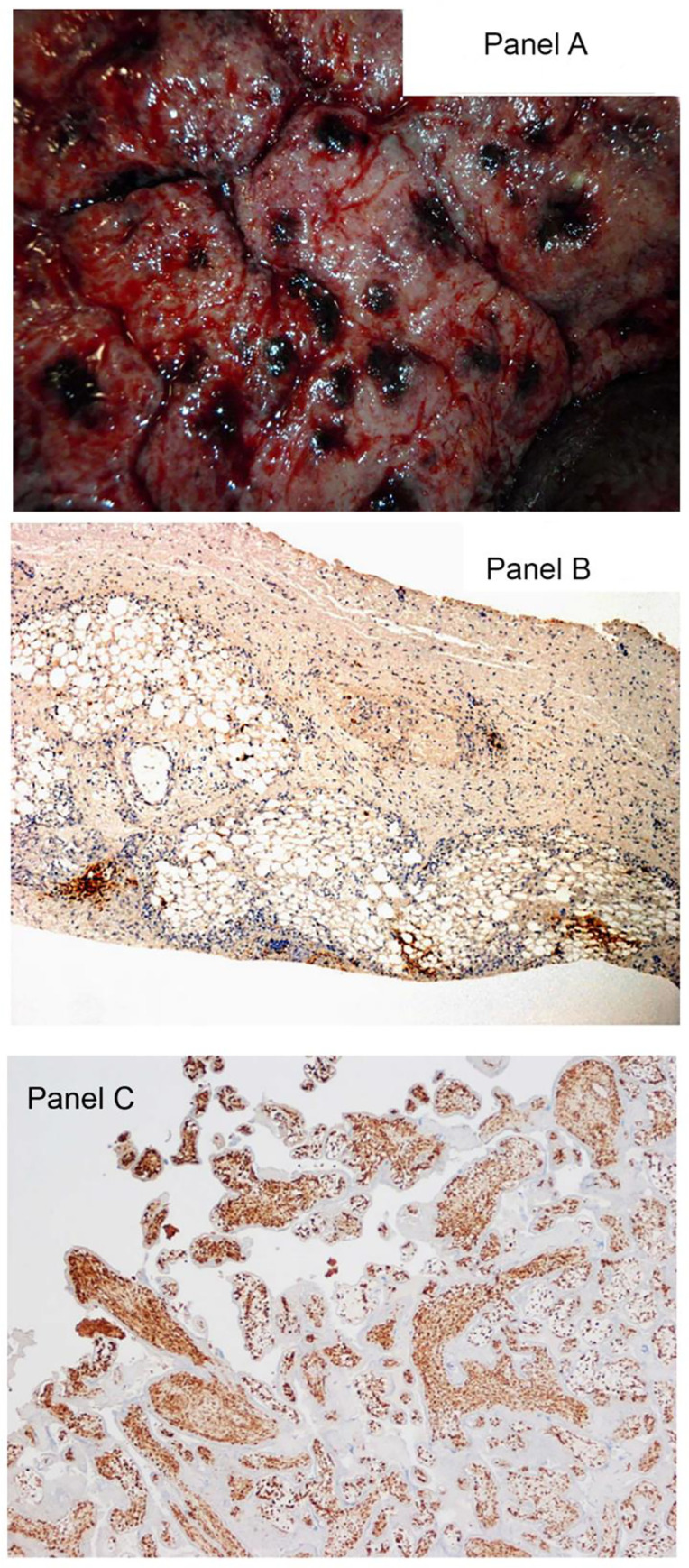
A. Maternal surface of the placenta with multiple grossly visible areas of basal hemorrhage. B. Fetal skin with multifocal positivity for virus using immunohistochemistry. Antibody to vaccinia virus counterstained with hematoxylin & eosin, X10. C. In the placenta immunohistochemistry demonstrates a pattern of intense and diffuse staining of chorionic villus stromal cells for MPXV that is consistent with macrophages (Hofbauer cells). The infected cells are increased in number, termed Hofbauer cell hyperplasia, a pattern that is seen in some TORCH viral infections. Antibody to vaccinia virus counterstained with hematoxylin & eosin, x4.

The course and outcome of four cases of maternal MPXV infection were described in a previous publication [30]. One of the four cases survived its mother’s mpox infection. Two pregnancies resulted in spontaneous abortions. The fourth had an intrauterine demise with a high viral load. PCR revealed a high viral load of MPXV in this fetus [30]. Examination of the placenta demonstrated numerous areas of hemorrhage involving the basal plate. Immunohistochemistry was performed using antibody to vaccinia virus, an orthopoxvirus which shares major antigens with MPXV. This showed a pattern of extensive and diffuse positive staining of chorionic villus stromal cells that were consistent with Hofbauer cells, the resident population of placental villous macrophages (Fig 7A–7C).

### Mpox disease deaths

Three deaths occurred among the 216 patients in this cohort for which a detailed accounting will be presented in a future publication. Two deaths occurred in the 5–11 year-old group, one in the <5 year-old group and none in the ≥12 year old age group. The 3 deaths are described briefly below:

**Fatal Case #1:** A gravely ill pediatric patient had 2091 lesions (VES 578, PUS 1375, OMB 37) on admission at 6^th^ day of fever and 5^th^ day of rash. The patient’s viral load by PCR was 2.4 X 10^6^ viral genomes/mL blood on admission. By day 2 lesions had increased to 2447 and fever had resolved. The patient developed respiratory distress and agonal breathing by 1700 and died at 2200 with elevated AST of 185 (2) and ALP of 304. Fig 7A–7C shows lesions on patient’s body, maternal surface of placenta and microscopic findings of placenta and infant skin lesions.
**Fatal Case #2:** A pediatric patient was admitted with complaints of fever for 3 days with onset of pustular lesions. More than 1,000 vesicular and pustular lesions, along with a few umbilicated lesions were observed. The clinical assessment was a child with severe early—stage mpox, dehydration, ketoacidosis and proteinuria. Mpox viral genomes in blood were initially 10^6^ genomes/mL and remained close to that value until death (this case will be reported in detail in a later manuscript).
**Fatal Case #3:** A third pediatric patient, part of a family cluster of 4 individuals was admitted on the 4^th^ day of fever and 3^rd^ day after lesions developed. The patient had a very high viral load (2 X 10^6^) on admission, 601 lesions and died the next day with very high liver transaminase values (AST = 865).
Death occurred in one pediatric patient from apparent respiratory illness 9 days after discharge from hospital with mpox at which time the patient was disease free. The death occurred at a location remote to the study site and investigators only became aware of the child’s death when the family did not return for the post-hospitalization follow-up visit. The cause of death cannot be confirmed as mpox. Although, orthopoxvirus induced immunosuppression is suspected as having contributed to the patient’s death by investigators. This case will be discussed in detail in a later publication.

### Association analyses

**Fig 8:**
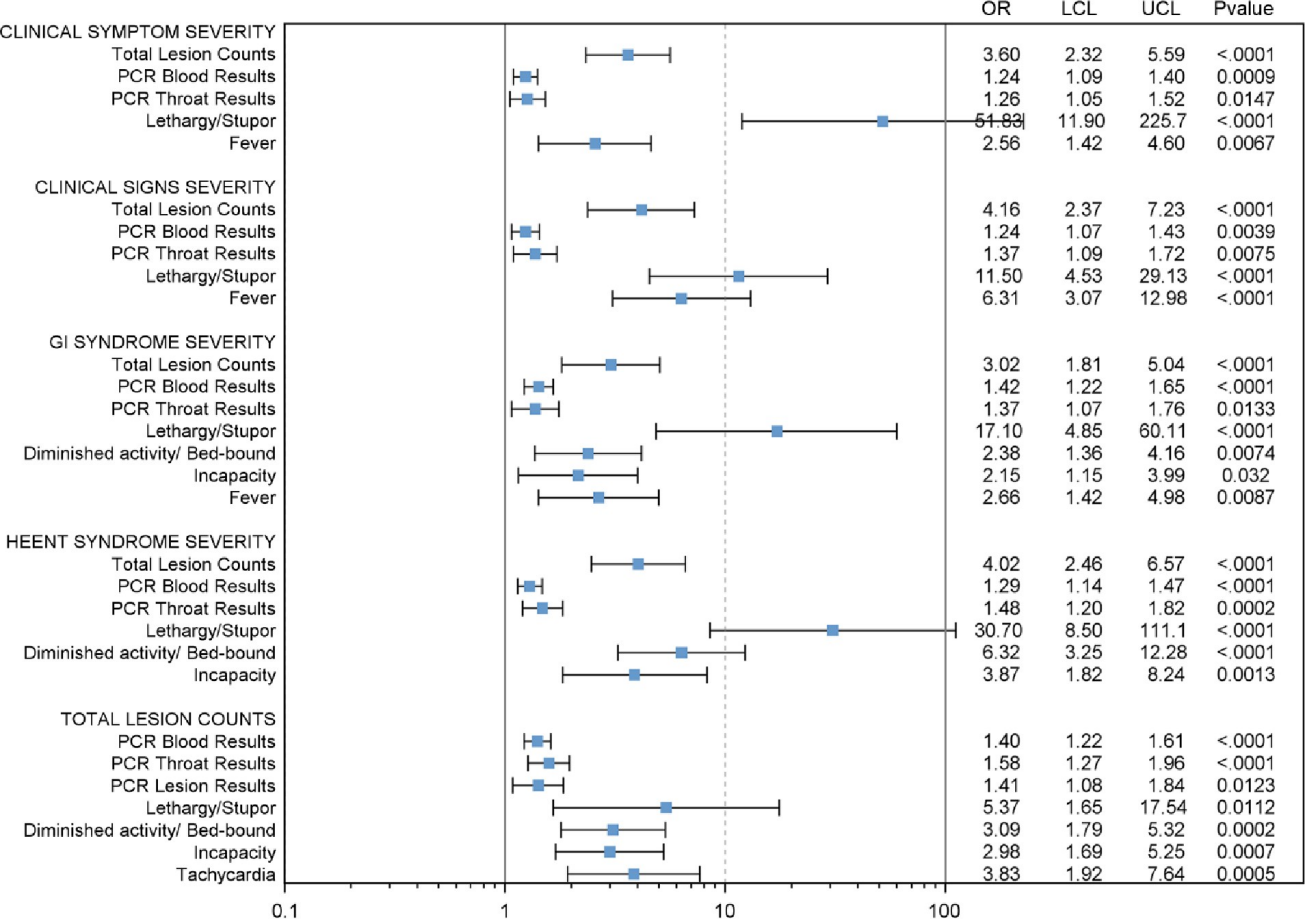
Forest Plot showing statistically significant associations. Odd ratio (OR), upper 95% confidence interval (UCL), and lower 95% confidence interval (LCL) were calculated using separate generalized estimating equations (GEE) with cumulative logit models, day after rash onset was adjusted as covariate. Adjusted *p* value were calculated using stepdown Bonferroni correction if needed. Only significant results are showed here (*p* value ≤ 0.05).

Forest Plot (Fig 8) shows statistically significant associations of total lesion count, PCR blood and throat values, neurologic findings, diminished activity, fever and tachycardia for gastrointestinal syndrome, HEEENT syndrome and lesion count severity. Neurologic signs (confused/disoriented/lethargy/stupor) have the highest Odd Ratios (ORs) relative to syndromic severity (ORs up to 23.30; P<0.0001); followed by activity level (Incapacity) with ORs up to 6.23. The association of other variables to total lesion count score severity, HEENT severity and GI severity are shown as additional Forest Plots in Supplemental materials (S1–S3 Figs).

## Discussion

The most complete early description of human MPX infection comes from Bremen’s description of 47 cases between 1970–1979 in West and Central Africa and Ježek’s 338 cases between 1981–1986 from the two WHO MPX study sites in Zaire (opened after the eradication of smallpox, reported in 1980) [31,32]. The epidemiologists described human mpox as closely resembling, discrete ordinary-type smallpox or occasionally modified type smallpox. No cases of human mpox have yet been reported that resemble either confluent or hemorrhagic smallpox either in the appearance of the exanthem or the rapidly fatal clinical course. The results of this investigation reinforce prior observations of the severity and systemic nature of this infection, while also highlighting opportunities to ameliorate clinical care and patient management.

All patients had one or more exposures-animal or household. Exposure to 2 or more wild animals was noted in 94.9% of patients (Table 1). Interpersonal transmission is most frequent in the ongoing world-wide outbreak [23,24]. In his series of human MPXD Girometti [24] reported that all of 54 individuals identified themselves as having sex with men. Bremen reported the rate of interhuman transmission to susceptible household members as being 7.5% in his series [31]. In this report, the rate of secondary household transmission to be 17.5%. Three-quarters (72.7% of households with an index case of mpox did not suffer secondary infections in our study.

Frequently occurring symptoms included sore throat, anorexia, cough, fever, chills, nasal discharge and congestion, dysphagia, mouth/throat lesions, headache, abdominal pain, sweats conjunctiva lesions, shortness of breath. Frequently occurring physical findings were classic MPXD rash, diminished levels of physical activity, nonspecific rash, mouth/throat lesions, fever, abnormal lung sounds, hepatomegaly/splenomegaly, lethargy/stupor, dehydration, confusion and or disorientation. Although we determined the duration of symptoms from the day of admission, the date of onset of symptoms and severity were not collected, thus the absolute duration and sequence of symptoms cannot be determined from this study. As a consequence of the low mortality rate, we were unable to draw firm conclusions regarding symptoms or physical findings that correlate with fatal infections. On a case-by-case basis, patients without these symptoms and signs could be considered for triage to home treatment, providing hygiene, hydration and nutritional support and infection control methods are available within the family unit. There is still much clinical work needed to refine and validate a grading system to provide decision support regarding disposition and treatment of individual patients. Any grading system should take the age of the patient into account as one of the major factors in determining the need and benefit of hospital-based care.

Wide variation was observed in lesion counts. The classic MPXV rash or lesion was observed in 99.5% of patients. The single patient with MPXD without lesions was determined to have been vaccinated against smallpox decades earlier. The lesion distribution is the classic centrifugal pattern as with smallpox (Fig 2) and correlated with disease severity. The lesion count was higher in cases of death, geometric mean = 2,294 compared to surviving patients geometric mean = 195; however, the low death rate does not allow testing for statistical significance.

There is no validated lesion count severity scoring system for MPXD. We used the CDC lesion count severity rating for smallpox to analyze symptoms and physical examination findings.

The most characteristic and distinguishing characteristic of mpox from smallpox has been the appearance of larger numbers of cases of mpox with painful, tender lymphadenopathy most frequently noted in the cervical and submandibular regions, but also occurring in inguinal regions or generalized. In this study, 98.6% of patients had lymphadenopathy which validates previous observations. Enlarged lymph nodes may compress the airway leading to respiratory compromise. Parental steroids may be indicated in these situations. Patients with multiple oropharyngeal lesions may refuse to eat or drink as well and may become dehydrated. Because motorized transportation was not available, many patients arrived at the hospital, dehydrated, malnourished, anorexic, and fatigued, adding to the physiologic stress of infection. Intensive nursing care and hydration would benefit these patients.

Because smallpox had largely disappeared from the developed world by the beginning of the 20^th^ Century, little is known or understood regarding extent of organ systems effected directly by VARV, or how isolated impairment of liver or renal function may have contributed to the morbidity or mortality of the illness. This study used routine hospital laboratory testing to evaluate the occurrence of organ system dysfunction and compare to clinical parameters of disease morbidity. Higher levels of ALT (mean = 90 vs 26) and AST (415 vs 48) were noted in fatal cases vs survivors, respectively but the low number of deaths makes statistical testing infeasible. Evidence of multisystem failure was only seen as a pre-terminal event.

PCR detected MPXV DNA in both blood and throat swabs before the onset of rash. The PCR viral load from the throat were about 2000 genomes/mL more than blood supporting the idea of swabbing the throat especially when MPXV infection is suspected but blood PCR values are low or negative. This finding could be relevant to the current mpox outbreak and early diagnosis.

As with smallpox, the scabs of MPXD are highly concentrated with virus when it separates. Although the infectiousness of the scab for MPXD is unknown, it is well documented that smallpox scabs are infectious and were used in biological events [33]. ACAM2000 vaccination is expected to form vesicles which result in a scab and characteristic scar. The ACAM2000 vaccination site remains positive for PCR DNA for at least two weeks after detachment of the scab [34]. This fact should be considered when advising patients about possible infectious nature and the duration of the scab site as well as the scab itself. These findings have implications for infection control as well.

Maximum PCR values are known to correlate with outcome in the lethal cynomolgus primate model including significant reductions with successfully antiviral drug treatment [7,29,30]. Maximum values occur early in the disease course which can be accurately determined in controlled animal studies; however, patients entered the study after rash onset at a point at which the peak viremia had passed resulting in the study failing to capture viral load peak levels for many patients. Thus, being admitted several days after rash onset reduced our ability to statistically validate maximum PCR with certain disease indicators (consistent with early admissions having higher maximum PCR values). Early disease seen in one patient supports the idea that maximum PCR values (a measure of viral load) occur very early in the disease course. Patients admitted several days after lesion onset are already past their maximum PCR value which explains why patients admitted closer to the onset of their rash tended to have higher maximum detected PCR values. For this study, the true maximum viral load was not determinable due to late admission of patients with MPXD.

Unlike PCR maximum values, which are transient, scarring from mpox lesions is more long-lasting. Thus, maximum lesion counts determined later in the course of the illness correlated with disease severity more clearly than observed maximum viral load. Nevertheless, patients who died had higher maximum PCR blood genome levels (geometric mean = 9,204,937, 95% CI: 2.1x10^4^–4.0x10^9^) compared to surviving patients (geometric mean = 22,971, 95% CI: 1.4x10^4^-3.8x10^4^). The low death rate prevents testing for statistical significance.

The case fatality rate of 1.4% (3/216) is significantly lower in this hospitalized cohort than has occurred historically. A case-fatality rate of 9.8% (33/338) was noted during a WHO sponsored study in Zaire during 1981–1986 [26]. A later study sponsored by the WHO during 1996–7 had a case-fatality rate of 3.7% [35]. The first case of human MPXD occurred in a child in 1970 who died from measles several days of recovering from MPXD (1). In this study, death of a pediatric patient occurred 9 days after being discharged from the hospital free from mpox disease. While being carried by its parents, the child developed an acute respiratory infection during the long journey home and died 3 days after arrival at their village.

Intrauterine fetal demise, the topic of a previous publication [30], occurred in 3 of 4 (75%) pregnancies due to maternal MPXV infection. The placenta of one fetus had a high level of MPXV infection shown by immunohistochemistry. The pattern of infected cells was predominantly within the chorionic villi, where the villous stromal cells demonstrated intense and confluent staining (Fig 7C). These stromal cells were consistent with Hofbauer cells, the native population of fetal macrophages within the chorionic villi. Hofbauer cells may produce inflammatory mediators and cytokines, damage the villus, and induce fibrosis as part of the inflammatory response to maternal infection [36]. These macrophages have been implicated in such other TORCH infections as Zika virus, SARS-CoV-2 and HIV [36,37,38]. Importantly, this pattern of staining is typical of maternal viruses that are transmitted hematogenously from the maternal bloodstream through the maternal-fetal interface at the level of the chorionic villi into the fetal circulation. This mechanism of intrauterine transmission has recently been suggested for MPXV [39].

Jynneos, a non-replicating, third generation vaccinia vaccine, was recently licensed for the prevention of smallpox and mpox [40,41]. Studies are underway to examine the efficacy of this vaccine in prevention of human MPX [42]. Additionally, Tecovirimat (TPOXX, ST-246) and Brincidofovir were recently licensed by FDA for the treatment of smallpox infection; however, FDA allows these medications to be used to treat individuals with mpox during the current outbreak of mpox infection [43,44,45]. As with other infectious diseases and intoxications, treatment before the onset of symptoms has the best outcomes.

## Supporting information

S1 Fig Associations between total lesion severity with other variables. Forest Plot showing associations between total lesion severity and other variables on the admission day. OR, UCL, LCL were calculated using GEE with cumulative logit models, day after rash onset and age group were adjusted as covariate and *p* value were adjusted by stepdown Bonferroni correction. The CDC lesion severity scale was used for classification of total lesion count: <25; 25–99; 10–499, ≥500.
(TIF)

S2 Fig Associations between HEENT syndrome severity with other variables. HEENT included following clinical symptoms or signs: visual changes, eye pain/discharge, ear pain, nasal discharge/congestion, dysphagia, = sore throat, conjunctive and other eye lesion, Nasal discharge/congestion/ rhinorrhea/nasal lesion, mouth/throat lesions. The HEENT severity scores are based on the number of abnormal HEENT: Grade 0 (0), Grade 1 (1–2), Grade 2 (3–5), Grade 3 (6–7), Grade 4 (8–9).
(TIF)

S3 Fig Associations between GI syndrome severity with other variables. GI included following clinical symptoms or signs: anorexia, vomiting, abdominal pain, dysphagia, diarrhea, hepatomegaly, splenomegaly or both, abdominal tenderness. The GI severity scores are based on the number of abnormal GI findings: Grade 0 (0), Grade 1 (1–2), Grade 2 (3–4), Grade 3 (5–8).
(TIF)

S1 Dataset Dataset 1 EXCEL. (XLS)

S2 Dataset Dataset 2 EXCEL. (XLS)

S3 Dataset Dataset 3 EXCEL. (XLS)

S1 Table CBC severity by age group. Each CBC component is graded as mild, moderate, severe or potentially life threatening for each age group and for the entire cohort. The CBC component was based on the most severe observation during the hospitalization.
(DOCX)

S2 Table Blood chemistry severity by age group. Blood chemistry tests are graded mild, moderate, severe or potentially life threatening by age group and for the entire cohort. The blood chemistry test grading is based upon the most severe observation during hospital course.
(DOCX)

S3 Table Urine severity by age group. Urinary glucose and protein grading as mild, moderate, severe or potentially life threatening is shown by age group and for the total cohort. The grading level is determined by the most severe observation during hospital course.
(DOCX)

S4 Table CBC grading by lesion count severity score. Each CBC component is graded as mild, moderate, severe or potentially life threatening for each lesion count range (lesion count severity score) on admission. The CBC component is graded based upon the most severe observation during hospitalization.
(DOCX)

S5 Table Blood chemistry severity by lesion count severity score. Each component of the blood chemistry panel is graded as mild, moderate, severe, or potentially life threatening for each lesion count range (lesion count severity score) on admission. Blood chemistry grading is based on the most severe observation during hospitalization.
(DOCX)

S6 Table Urine severity by lesion count severity score. Urinary glucose and protein are graded mild, moderate, severe, or potentially life threatening for each lesion count range (lesion count severity score) on admission. Urinary glucose and protein are based on the most severe observation during hospitalization.
(DOCX)
