# Supplementary figures and images for: Clinical characterization and placental pathology of mpox infection in hospitalized patients in the Democratic Republic of the Congo
Source: PLoS Negl Trop Dis. 2023 Apr 20;17(4):e0010384. doi: 10.1371/journal.pntd.0010384 (PMC10153724; doi:10.1371/journal.pntd.0010384)

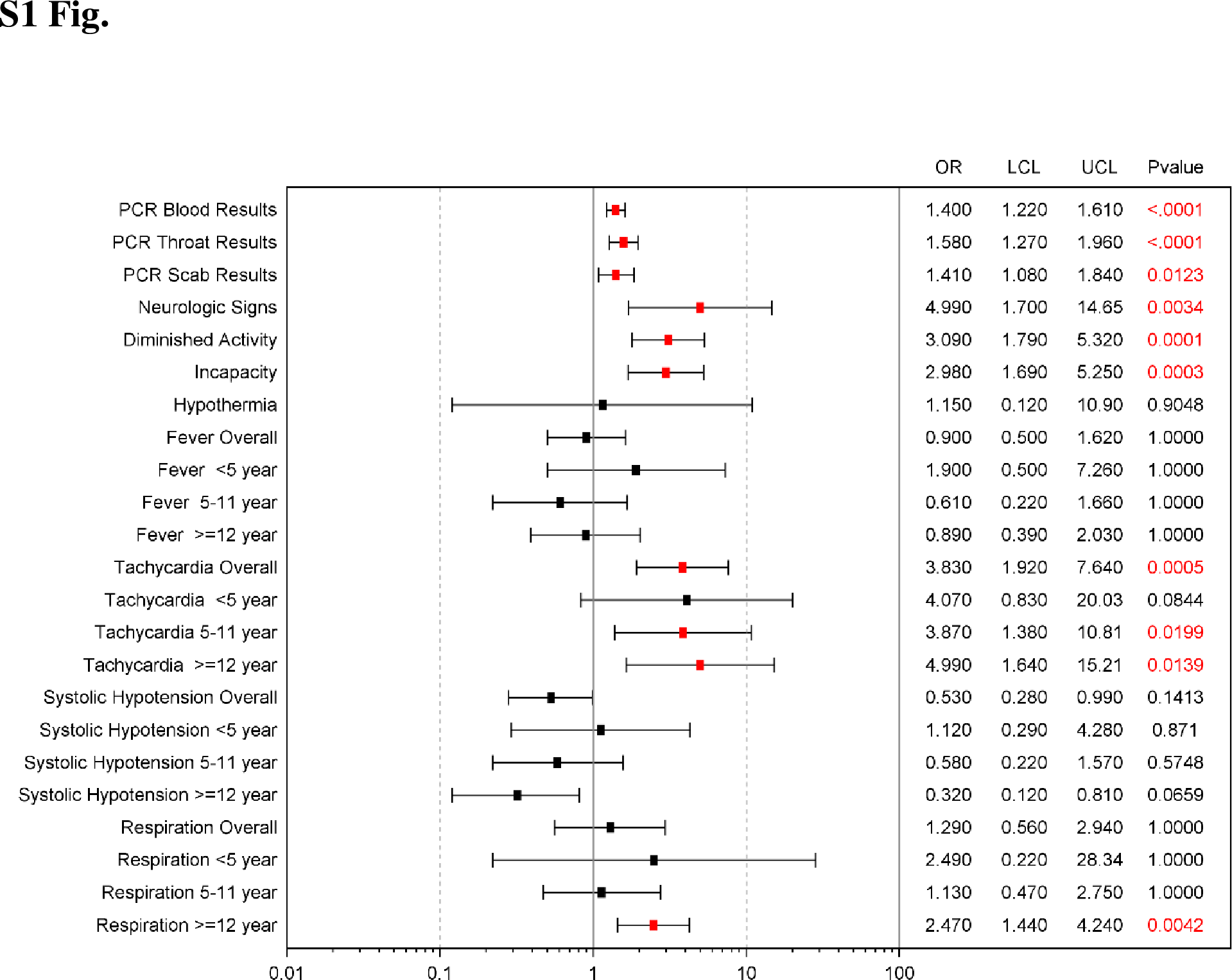

Supplement: S1 Fig — Forest Plot showing associations between total lesion severity and other variables on the admission day. OR, UCL, LCL were calculated using GEE with cumulative logit models, day after rash onset and age group were adjusted as covariate and p value were adjusted by stepdown Bonferroni correction. The CDC lesion severity scale was used for classification of total lesion count: <25; 25–99; 10–499, ≥500. (TIF) [file pntd.0010384.s001.tif]

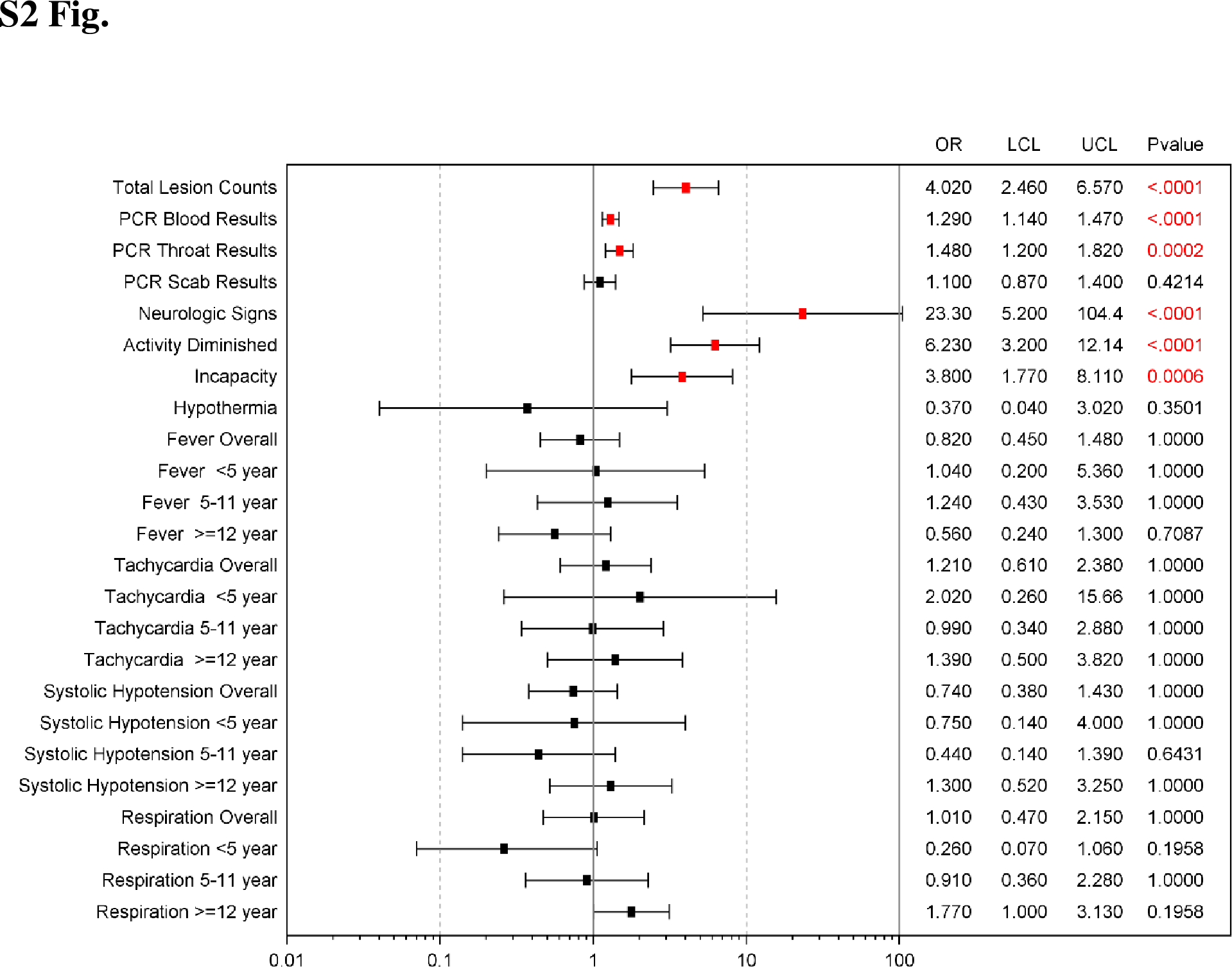

Supplement: S2 Fig — HEENT included following clinical symptoms or signs: visual changes, eye pain/discharge, ear pain, nasal discharge/congestion, dysphagia, = sore throat, conjunctive and other eye lesion, Nasal discharge/congestion/ rhinorrhea/nasal lesion, mouth/throat lesions. The HEENT severity scores are based on the number of abnormal HEENT: Grade 0 (0), Grade 1 (1–2), Grade 2 (3–5), Grade 3 (6–7), Grade 4 (8–9). (TIF) [file pntd.0010384.s002.tif]

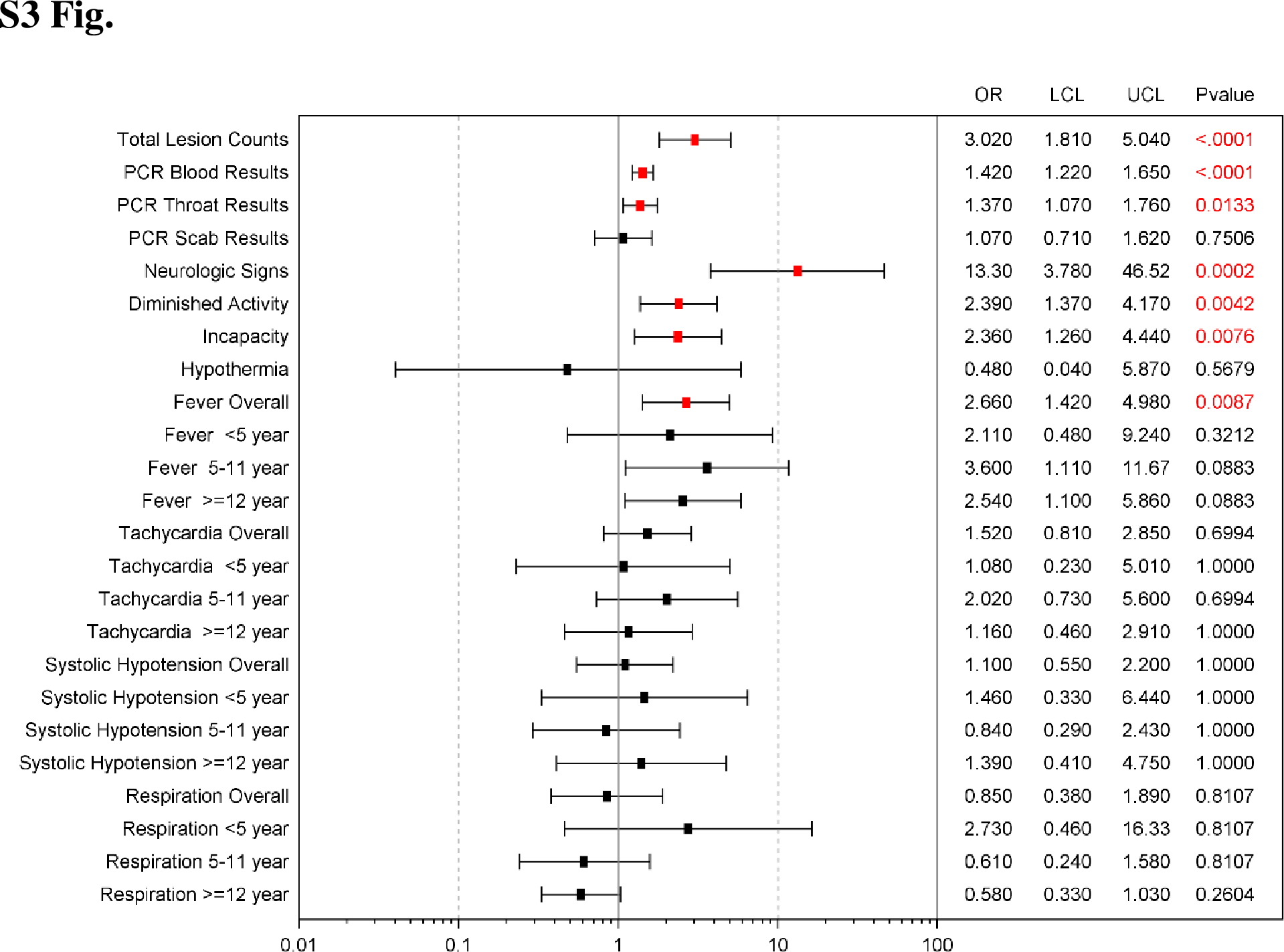

Supplement: S3 Fig — GI included following clinical symptoms or signs: anorexia, vomiting, abdominal pain, dysphagia, diarrhea, hepatomegaly, splenomegaly or both, abdominal tenderness. The GI severity scores are based on the number of abnormal GI findings: Grade 0 (0), Grade 1 (1–2), Grade 2 (3–4), Grade 3 (5–8). (TIF) [file pntd.0010384.s003.tif]
