# Supplementary material for: Clinical characterization and placental pathology of mpox infection in hospitalized patients in the Democratic Republic of the Congo
Source: PLoS Negl Trop Dis. 2023 Apr 20;17(4):e0010384. doi: 10.1371/journal.pntd.0010384 (PMC10153724; doi:10.1371/journal.pntd.0010384)
Supplement: S1 Table — Each CBC component is graded as mild, moderate, severe or potentially life threatening for each age group and for the entire cohort. The CBC component was based on the most severe observation during the hospitalization. (DOCX) [file pntd.0010384.s007.docx]

**S1 Table. CBC severity by age group.**

|  | | | **Age Group** | | | |  | |
| --- | --- | --- | --- | --- | --- | --- | --- | --- |
|  |  | **<5 (N=31)** | | **5-11 (N=67)** | **≥ 12 (N=118)** | **Total (N=216)** | |  |
| **Laboratory Test (Unit)** | **Severity** | **n (%)** | | **n (%)** | **n (%)** | **n (%)** | |  |
| Hg (gm/dL) | Mild | 1 (3.2) | | 2 (3.0) | 22 (18.6) | 25 (11.6) | |  |
|  | Moderate | 3 (9.7) | | 13 (19.4) | 24 (20.3) | 40 (18.5) | |  |
|  | Severe | 7 (22.6) | | 28 (41.8) | 26 (22.0) | 61 (28.2) | |  |
|  | Potentially Life Threatening | 11 (35.5) | | 13 (19.4) | 12 (10.2) | 36 (16.7) | |  |
|  | | | | | | | | |
| WBC increase (10^3 cell/) | Mild | 7 (22.6) | | 24 (35.8) | 34 (28.8) | 65 (30.1) | |  |
|  | Moderate | 3 (9.7) | | 13 (19.4) | 12 (10.2) | 28 (13.0) | |  |
|  | Severe | 5 (16.1) | | 6 (9.0) | 3 (2.5) | 14 (6.5) | |  |
|  | Potentially Life Threatening | 10 (32.3) | | 0 (0.0) | 1 (0.8) | 11 (5.1) | |  |
|  | | | | | | | | |
| WBC decrease (10^3 cell/) | Mild | 0 (0.0) | | 7 (10.4) | 7 (5.9) | 14 (6.5) | |  |
|  | Moderate | 0 (0.0) | | 1 (1.5) | 2 (1.7) | 3 (1.4) | |  |
|  | Severe | 0 (0.0) | | 0 (0.0) | 1 (0.8) | 1 (0.5) | |  |
|  | Potentially Life Threatening | 0 (0.0) | | 0 (0.0) | 1 (0.8) | 1 (0.5) | |  |
|  | | | | | | | | |
| Neut decrease (cell/mm^3) | Mild | 3 (9.7) | | 14 (20.9) | 15 (12.7) | 32 (14.8) | |  |
|  | Moderate | 6 (19.4) | | 6 (9.0) | 23 (19.5) | 35 (16.2) | |  |
|  | Severe | 2 (6.5) | | 6 (9.0) | 12 (10.2) | 20 (9.3) | |  |
|  | Potentially Life Threatening | 4 (12.9) | | 8 (11.9) | 20 (16.9) | 32 (14.8) | |  |
|  | | | | | | | | |
| Lymph decrease (Lymph + Atypical) (cell/mm^3) | Mild | 0 (0.0) | | 0 (0.0) | 0 (0.0) | 0 (0.0) | |  |
|  | Moderate | 0 (0.0) | | 0 (0.0) | 1 (0.8) | 1 (0.5) | |  |
|  | Severe | 0 (0.0) | | 0 (0.0) | 0 (0.0) | 0 (0.0) | |  |
|  | Potentially Life Threatening | 0 (0.0) | | 1 (1.5) | 2 (1.7) | 3 (1.4) | |  |
|  | | | | | | | | |
| EOSIN (cell/mm^3) | Mild | 10 (32.3) | | 23 (34.3) | 34 (28.8) | 67 (31.0) | |  |
|  | Moderate | 13 (41.9) | | 36 (53.7) | 72 (61.0) | 121 (56.0) | |  |
|  | Severe | 5 (16.1) | | 3 (4.5) | 4 (3.4) | 12 (5.6) | |  |
|  |  |  | |  |  |  | |  |
| PLT decrease (10^3/UL) | Mild | 1 (3.2) | | 5 (7.5) | 22 (18.6) | 28 (13.0) | |  |
|  | Moderate | 2 (6.5) | | 4 (6.0) | 13 (11.0) | 19 (8.8) | |  |
|  | Severe | 2 (6.5) | | 5 (7.5) | 5 (4.2) | 12 (5.6) | |  |
|  | Potentially Life Threatening | 1 (3.2) | | 3 (4.5) | 2 (1.7) | 6 (2.8) | |  |

Laboratory test severity grade based on most severe observation during hospitalization.
