# Supplementary material for: Clinical characterization and placental pathology of mpox infection in hospitalized patients in the Democratic Republic of the Congo
Source: PLoS Negl Trop Dis. 2023 Apr 20;17(4):e0010384. doi: 10.1371/journal.pntd.0010384 (PMC10153724; doi:10.1371/journal.pntd.0010384)
Supplement: S2 Table — Blood chemistry tests are graded mild, moderate, severe or potentially life threatening by age group and for the entire cohort. The blood chemistry test grading is based upon the most severe observation during hospital course. (DOCX) [file pntd.0010384.s008.docx]

**S2 Table: Blood chemistry severity by age group.**

|  | | **Age Group** | | |  |
| --- | --- | --- | --- | --- | --- |
|  |  | **<5 (N=31)** | **5-11 (N=67)** | **≥ 12 (N=118)** | **Total (N=216)** |
| **Laboratory Test (Unit)** | **Severity** | **n (%)** | **n (%)** | **n (%)** | **n (%)** |
| ALT (U/L) | Mild | 3 (9.7) | 11 (16.4) | 34 (28.8) | 48 (22.2) |
|  | Moderate | 1 (3.2) | 3 (4.5) | 10 (8.5) | 14 (6.5) |
|  | Severe | 0 (0.0) | 0 (0.0) | 0 (0.0) | 0 (0.0) |
|  | Potentially Life Threatening | 0 (0.0) | 1 (1.5) | 0 (0.0) | 1 (0.5) |
|  | | | | | |
| AST (U/L) | Mild | 13 (41.9) | 35 (52.2) | 65 (55.1) | 113 (52.3) |
|  | Moderate | 3 (9.7) | 9 (13.4) | 28 (23.7) | 40 (18.5) |
|  | Severe | 1 (3.2) | 2 (3.0) | 2 (1.7) | 5 (2.3) |
|  | Potentially Life Threatening | 1 (3.2) | 3 (4.5) | 0 (0.0) | 4 (1.9) |
|  | | | | | |
| ALB (hypoalbuminemia) (G/DL) | Mild | 1 (3.2) | 3 (4.5) | 7 (5.9) | 11 (5.1) |
|  | Moderate | 23 (74.2) | 51 (76.1) | 106 (89.8) | 180 (83.3) |
|  | Severe | 5 (16.1) | 13 (19.4) | 4 (3.4) | 22 (10.2) |
|  | | | | | |
| ALP (U/L) | Mild | 6 (19.4) | 11 (16.4) | 25 (21.2) | 42 (19.4) |
|  | Moderate | 1 (3.2) | 0 (0.0) | 0 (0.0) | 1 (0.5) |
|  | Severe | 0 (0.0) | 0 (0.0) | 0 (0.0) | 0 (0.0) |
|  | Potentially Life Threatening | 0 (0.0) | 0 (0.0) | 0 (0.0) | 0 (0.0) |
|  | | | | | |
| AMY (U/L) | Mild | 9 (29.0) | 15 (22.4) | 40 (33.9) | 64 (29.6) |
|  | Moderate | 4 (12.9) | 16 (23.9) | 24 (20.3) | 44 (20.4) |
|  | Severe | 4 (12.9) | 14 (20.9) | 11 (9.3) | 29 (13.4) |
|  | Potentially Life Threatening | 1 (3.2) | 2 (3.0) | 0 (0.0) | 3 (1.4) |
|  | | | | | |
| BUN (MG/DL) | Mild | 0 (0.0) | 0 (0.0) | 1 (0.8) | 1 (0.5) |
|  | Moderate | 0 (0.0) | 0 (0.0) | 2 (1.7) | 2 (0.9) |
|  | Severe | 0 (0.0) | 0 (0.0) | 2 (1.7) | 2 (0.9) |
|  | | | | | |
| CA (hypocalcemia) (MG/DL) | Mild | 5 (16.1) | 18 (26.9) | 0 (0.0) | 23 (10.6) |
|  | Moderate | 2 (6.5) | 5 (7.5) | 7 (5.9) | 14 (6.5) |
|  | Severe | 2 (6.5) | 1 (1.5) | 1 (0.8) | 4 (1.9) |
|  | Potentially Life Threatening | 0 (0.0) | 3 (4.5) | 4 (3.4) | 7 (3.2) |
|  | | | | | |
| CA (hypercalcemia) (MG/DL) | Mild | 0 (0.0) | 0 (0.0) | 0 (0.0) | 0 (0.0) |
|  | Moderate | 0 (0.0) | 0 (0.0) | 0 (0.0) | 0 (0.0) |
|  | Severe | 0 (0.0) | 0 (0.0) | 0 (0.0) | 0 (0.0) |
|  | Potentially Life Threatening | 0 (0.0) | 0 (0.0) | 0 (0.0) | 0 (0.0) |
|  | | | | | |
| CRE (MG/DL) | Mild | 0 (0.0) | 1 (1.5) | 1 (0.8) | 2 (0.9) |
|  | Moderate | 0 (0.0) | 0 (0.0) | 0 (0.0) | 0 (0.0) |
|  | Severe | 0 (0.0) | 0 (0.0) | 0 (0.0) | 0 (0.0) |
|  | Potentially Life Threatening | 0 (0.0) | 1 (1.5) | 1 (0.8) | 2 (0.9) |
|  | | | | | |
| GLU (hypoglycemia) (MG/DL) | Mild | 4 (12.9) | 12 (17.9) | 10 (8.5) | 26 (12.0) |
|  | Moderate | 2 (6.5) | 4 (6.0) | 7 (5.9) | 13 (6.0) |
|  | Severe | 0 (0.0) | 0 (0.0) | 2 (1.7) | 2 (0.9) |
|  | Potentially Life Threatening | 0 (0.0) | 0 (0.0) | 1 (0.8) | 1 (0.5) |
|  | | | | | |
| GLU (hyperglycemia) (MG/DL) | Mild | 6 (19.4) | 7 (10.4) | 21 (17.8) | 34 (15.7) |
|  | Moderate | 0 (0.0) | 1 (1.5) | 4 (3.4) | 5 (2.3) |
|  | Severe | 0 (0.0) | 0 (0.0) | 0 (0.0) | 0 (0.0) |
|  | Potentially Life Threatening | 0 (0.0) | 0 (0.0) | 0 (0.0) | 0 (0.0) |
|  | | | | | |
| TBIL (normal LFT) (MG/DL) | Mild | 1 (3.2) | 1 (1.5) | 0 (0.0) | 2 (0.9) |
|  | Moderate | 1 (3.2) | 0 (0.0) | 0 (0.0) | 1 (0.5) |
|  | Severe | 1 (3.2) | 0 (0.0) | 0 (0.0) | 1 (0.5) |
|  | Potentially Life Threatening | 0 (0.0) | 0 (0.0) | 2 (1.7) | 2 (0.9) |
|  | | | | | |
| TP (Hypoproteinemia) (G/DL) | Mild | 2 (6.5) | 3 (4.5) | 1 (0.8) | 6 (2.8) |
|  | Moderate | 1 (3.2) | 0 (0.0) | 0 (0.0) | 1 (0.5) |
|  | Severe | 1 (3.2) | 1 (1.5) | 3 (2.5) | 5 (2.3) |
|  | | | | | |
| GGT (U/L) | Mild | 8 (25.8) | 23 (34.3) | 27 (22.9) | 58 (26.9) |
|  | Moderate | 1 (3.2) | 4 (6.0) | 5 (4.2) | 10 (4.6) |
|  | Severe | 2 (6.5) | 1 (1.5) | 0 (0.0) | 3 (1.4) |
|  | Potentially Life Threatening | 1 (3.2) | 0 (0.0) | 0 (0.0) | 1 (0.5) |

Laboratory test severity grade based on most severe observation during hospitalization.
