# Supplementary material for: Clinical characterization and placental pathology of mpox infection in hospitalized patients in the Democratic Republic of the Congo
Source: PLoS Negl Trop Dis. 2023 Apr 20;17(4):e0010384. doi: 10.1371/journal.pntd.0010384 (PMC10153724; doi:10.1371/journal.pntd.0010384)
Supplement: S3 Table — Urinary glucose and protein grading as mild, moderate, severe or potentially life threatening is shown by age group and for the total cohort. The grading level is determined by the most severe observation during hospital course. (DOCX) [file pntd.0010384.s009.docx]

**S3 Table: Urine severity by age group.**

|  | | **Age Group** | | |  |
| --- | --- | --- | --- | --- | --- |
|  |  | **<5 (N=31)** | **5-11 (N=67)** | **≥ 12 (N=118)** | **Total (N=216)** |
| **Laboratory Test** | **Severity** | **n (%)** | **n (%)** | **n (%)** | **n (%)** |
| Glucose | Mild | 0 (0.0) | 3 (4.5) | 1 (0.8) | 4 (1.9) |
|  | Moderate | 2 (6.5) | 7 (10.4) | 27 (22.9) | 36 (16.7) |
|  | Severe | 0 (0.0) | 0 (0.0) | 1 (0.8) | 1 (0.5) |
|  | Potentially Life Threatening | 0 (0.0) | 0 (0.0) | 0 (0.0) | 0 (0.0) |
|  | | | | | |
| Protein | Mild | 2 (6.5) | 7 (10.4) | 11 (9.3) | 20 (9.3) |
|  | Moderate | 12 (38.7) | 18 (26.9) | 31 (26.3) | 61 (28.2) |
|  | Severe | 14 (45.2) | 30 (44.8) | 57 (48.3) | 101 (46.8) |
|  | Potentially Life Threatening | 1 (3.2) | 9 (13.4) | 19 (16.1) | 29 (13.4) |

Laboratory test severity grade based on most severe observation during hospitalization.
