# Supplementary material for: Clinical characterization and placental pathology of mpox infection in hospitalized patients in the Democratic Republic of the Congo
Source: PLoS Negl Trop Dis. 2023 Apr 20;17(4):e0010384. doi: 10.1371/journal.pntd.0010384 (PMC10153724; doi:10.1371/journal.pntd.0010384)
Supplement: S4 Table — Each CBC component is graded as mild, moderate, severe or potentially life threatening for each lesion count range (lesion count severity score) on admission. The CBC component is graded based upon the most severe observation during hospitalization. (DOCX) [file pntd.0010384.s010.docx]

**S4 Table: CBC severity by lesion severity score.**

|  | | | **Total Lesion Severity Score** | | | | |
| --- | --- | --- | --- | --- | --- | --- | --- |
|  |  | **<25 (N=20)** | | **25-99 (N=58)** | **100-499 (N=91)** | **≥500 (N=47)** |  |
| **Laboratory Test (Unit)** | **Severity** | **n (%)** | | **n (%)** | **n (%)** | **n (%)** |  |
| Hg (gm/dL) | Mild | 4 (20.0) | | 7 (12.1) | 11 (12.1) | 3 (6.4) |  |
|  | Moderate | 3 (15.0) | | 13 (22.4) | 15 (16.5) | 9 (19.1) |  |
|  | Severe | 5 (25.0) | | 16 (27.6) | 29 (31.9) | 11 (23.4) |  |
|  | Potentially Life Threatening | 4 (20.0) | | 10 (17.2) | 14 (15.4) | 8 (17.0) |  |
|  | | | | | | | |
| WBC increase (10^3 cell/) | Mild | 5 (25.0) | | 14 (24.1) | 31 (34.1) | 15 (31.9) |  |
|  | Moderate | 3 (15.0) | | 7 (12.1) | 10 (11.0) | 8 (17.0) |  |
|  | Severe | 1 (5.0) | | 2 (3.4) | 3 (3.3) | 8 (17.0) |  |
|  | Potentially Life Threatening | 0 (0.0) | | 1 (1.7) | 5 (5.5) | 5 (10.6) |  |
|  | | | | | | | |
| WBC decrease (10^3 cell/) | Mild | 2 (10.0) | | 4 (6.9) | 4 (4.4) | 4 (8.5) |  |
|  | Moderate | 2 (10.0) | | 0 (0.0) | 1 (1.1) | 0 (0.0) |  |
|  | Severe | 0 (0.0) | | 0 (0.0) | 1 (1.1) | 0 (0.0) |  |
|  | Potentially Life Threatening | 1 (5.0) | | 0 (0.0) | 0 (0.0) | 0 (0.0) |  |
|  | | | | | | | |
| Neut decrease (cell/mm^3) | Mild | 2 (10.0) | | 13 (22.4) | 13 (14.3) | 4 (8.5) |  |
|  | Moderate | 2 (10.0) | | 14 (24.1) | 13 (14.3) | 6 (12.8) |  |
|  | Severe | 4 (20.0) | | 6 (10.3) | 9 (9.9) | 1 (2.1) |  |
|  | Potentially Life Threatening | 7 (35.0) | | 9 (15.5) | 9 (9.9) | 7 (14.9) |  |
|  | | | | | | | |
| Lymph decrease (Lymph + Atypical) (cell/mm^3) | Mild | 0 (0.0) | | 0 (0.0) | 0 (0.0) | 0 (0.0) |  |
|  | Moderate | 0 (0.0) | | 0 (0.0) | 1 (1.1) | 0 (0.0) |  |
|  | Severe | 0 (0.0) | | 0 (0.0) | 0 (0.0) | 0 (0.0) |  |
|  | Potentially Life Threatening | 1 (5.0) | | 1 (1.7) | 1 (1.1) | 0 (0.0) |  |
|  | | | | | | | |
| EOSIN (cell/mm^3) | Mild | 6 (30.0) | | 15 (25.9) | 30 (33.0) | 16 (34.0) |  |
|  | Moderate | 12 (60.0) | | 36 (62.1) | 49 (53.8) | 24 (51.1) |  |
|  | Severe | 1 (5.0) | | 4 (6.9) | 4 (4.4) | 3 (6.4) |  |
|  |  |  | |  |  |  |  |
| PLT decrease (10^3/UL) | Mild | 2 (10.0) | | 7 (12.1) | 10 (11.0) | 9 (19.1) |  |
|  | Moderate | 3 (15.0) | | 2 (3.4) | 9 (9.9) | 5 (10.6) |  |
|  | Severe | 1 (5.0) | | 6 (10.3) | 5 (5.5) | 0 (0.0) |  |
|  | Potentially Life Threatening | 2 (10.0) | | 0 (0.0) | 2 (2.2) | 2 (4.3) |  |

Laboratory test severity grade based on most severe observation during hospitalization. Total lesion severity score equals total number of lesions present on admission day.
