# Supplementary material for: Clinical characterization and placental pathology of mpox infection in hospitalized patients in the Democratic Republic of the Congo
Source: PLoS Negl Trop Dis. 2023 Apr 20;17(4):e0010384. doi: 10.1371/journal.pntd.0010384 (PMC10153724; doi:10.1371/journal.pntd.0010384)
Supplement: S5 Table — Each component of the blood chemistry panel is graded as mild, moderate, severe, or potentially life threatening for each lesion count range (lesion count severity score) on admission. Blood chemistry grading is based on the most severe observation during hospitalization. (DOCX) [file pntd.0010384.s011.docx]

**S5 Table: Blood chemistry severity by lesion severity score.**

|  | | **Total Lesion Severity Score** | | | |
| --- | --- | --- | --- | --- | --- |
|  |  | **<25 (N=20)** | **25-99 (N=58)** | **100-499 (N=91)** | **≥500 (N=47)** |
| **Laboratory Test (Unit)** | **Severity** | **n (%)** | **n (%)** | **n (%)** | **n (%)** |
| ALT (U/L) | Mild | 3 (15.0) | 13 (22.4) | 22 (24.2) | 10 (21.3) |
|  | Moderate | 1 (5.0) | 2 (3.4) | 6 (6.6) | 5 (10.6) |
|  | Severe | 0 (0.0) | 0 (0.0) | 0 (0.0) | 0 (0.0) |
|  | Potentially Life Threatening | 0 (0.0) | 1 (1.7) | 0 (0.0) | 0 (0.0) |
|  | | | | | |
| AST (U/L) | Mild | 11 (55.0) | 24 (41.4) | 53 (58.2) | 25 (53.2) |
|  | Moderate | 3 (15.0) | 12 (20.7) | 16 (17.6) | 9 (19.1) |
|  | Severe | 0 (0.0) | 2 (3.4) | 3 (3.3) | 0 (0.0) |
|  | Potentially Life Threatening | 0 (0.0) | 1 (1.7) | 0 (0.0) | 3 (6.4) |
|  | | | | | |
| ALB (hypoalbuminemia) (G/DL) | Mild | 1 (5.0) | 5 (8.6) | 4 (4.4) | 1 (2.1) |
|  | Moderate | 19 (95.0) | 47 (81.0) | 77 (84.6) | 37 (78.7) |
|  | Severe | 0 (0.0) | 5 (8.6) | 9 (9.9) | 8 (17.0) |
|  | | | | | |
| ALP (U/L) | Mild | 4 (20.0) | 13 (22.4) | 19 (20.9) | 6 (12.8) |
|  | Moderate | 0 (0.0) | 1 (1.7) | 0 (0.0) | 0 (0.0) |
|  | Severe | 0 (0.0) | 0 (0.0) | 0 (0.0) | 0 (0.0) |
|  | Potentially Life Threatening | 0 (0.0) | 0 (0.0) | 0 (0.0) | 0 (0.0) |
|  | | | | | |
| AMY (U/L) | Mild | 3 (15.0) | 22 (37.9) | 22 (24.2) | 17 (36.2) |
|  | Moderate | 7 (35.0) | 10 (17.2) | 22 (24.2) | 5 (10.6) |
|  | Severe | 2 (10.0) | 3 (5.2) | 16 (17.6) | 8 (17.0) |
|  | Potentially Life Threatening | 0 (0.0) | 0 (0.0) | 3 (3.3) | 0 (0.0) |
|  | | | | | |
| BUN (MG/DL) | Mild | 0 (0.0) | 0 (0.0) | 0 (0.0) | 1 (2.1) |
|  | Moderate | 0 (0.0) | 0 (0.0) | 0 (0.0) | 2 (4.3) |
|  | Severe | 0 (0.0) | 0 (0.0) | 1 (1.1) | 1 (2.1) |
|  | | | | | |
| CA (hypocalcemia) (MG/DL) | Mild | 1 (5.0) | 5 (8.6) | 12 (13.2) | 5 (10.6) |
|  | Moderate | 1 (5.0) | 2 (3.4) | 7 (7.7) | 4 (8.5) |
|  | Severe | 0 (0.0) | 1 (1.7) | 1 (1.1) | 2 (4.3) |
|  | Potentially Life Threatening | 2 (10.0) | 2 (3.4) | 2 (2.2) | 1 (2.1) |
|  | | | | | |
| CA (hypercalcemia) (MG/DL) | Mild | 0 (0.0) | 0 (0.0) | 0 (0.0) | 0 (0.0) |
|  | Moderate | 0 (0.0) | 0 (0.0) | 0 (0.0) | 0 (0.0) |
|  | Severe | 0 (0.0) | 0 (0.0) | 0 (0.0) | 0 (0.0) |
|  | Potentially Life Threatening | 0 (0.0) | 0 (0.0) | 0 (0.0) | 0 (0.0) |
|  | | | | | |
| CRE (MG/DL) | Mild | 1 (5.0) | 0 (0.0) | 0 (0.0) | 1 (2.1) |
|  | Moderate | 0 (0.0) | 0 (0.0) | 0 (0.0) | 0 (0.0) |
|  | Severe | 0 (0.0) | 0 (0.0) | 0 (0.0) | 0 (0.0) |
|  | Potentially Life Threatening | 1 (5.0) | 0 (0.0) | 1 (1.1) | 0 (0.0) |
|  | | | | | |
| GLU (hypoglycemia) (MG/DL) | Mild | 3 (15.0) | 4 (6.9) | 13 (14.3) | 6 (12.8) |
|  | Moderate | 0 (0.0) | 4 (6.9) | 7 (7.7) | 2 (4.3) |
|  | Severe | 0 (0.0) | 1 (1.7) | 0 (0.0) | 1 (2.1) |
|  | Potentially Life Threatening | 0 (0.0) | 0 (0.0) | 1 (1.1) | 0 (0.0) |
|  | | | | | |
| GLU (hyperglycemia) (MG/DL) | Mild | 1 (5.0) | 6 (10.3) | 15 (16.5) | 12 (25.5) |
|  | Moderate | 0 (0.0) | 0 (0.0) | 3 (3.3) | 2 (4.3) |
|  | Severe | 0 (0.0) | 0 (0.0) | 0 (0.0) | 0 (0.0) |
|  | Potentially Life Threatening | 0 (0.0) | 0 (0.0) | 0 (0.0) | 0 (0.0) |
|  | | | | | |
| TBIL (normal LFT) (MG/DL) | Mild | 0 (0.0) | 0 (0.0) | 1 (1.1) | 1 (2.1) |
|  | Moderate | 0 (0.0) | 0 (0.0) | 0 (0.0) | 1 (2.1) |
|  | Severe | 0 (0.0) | 0 (0.0) | 1 (1.1) | 0 (0.0) |
|  | Potentially Life Threatening | 1 (5.0) | 0 (0.0) | 1 (1.1) | 0 (0.0) |
|  | | | | | |
| TP (Hypoproteinemia) (G/DL) | Mild | 0 (0.0) | 2 (3.4) | 1 (1.1) | 3 (6.4) |
|  | Moderate | 0 (0.0) | 0 (0.0) | 1 (1.1) | 0 (0.0) |
|  | Severe | 1 (5.0) | 1 (1.7) | 1 (1.1) | 2 (4.3) |
|  | | | | | |
| GGT (U/L) | Mild | 7 (35.0) | 10 (17.2) | 24 (26.4) | 17 (36.2) |
|  | Moderate | 0 (0.0) | 3 (5.2) | 3 (3.3) | 4 (8.5) |
|  | Severe | 0 (0.0) | 0 (0.0) | 1 (1.1) | 2 (4.3) |
|  | Potentially Life Threatening | 0 (0.0) | 0 (0.0) | 1 (1.1) | 0 (0.0) |

Laboratory test severity grade based on most severe observation during hospitalization. Total lesion severity score equals total number of

lesions present on admission day.
