# Supplementary material for: Clinical characterization and placental pathology of mpox infection in hospitalized patients in the Democratic Republic of the Congo
Source: PLoS Negl Trop Dis. 2023 Apr 20;17(4):e0010384. doi: 10.1371/journal.pntd.0010384 (PMC10153724; doi:10.1371/journal.pntd.0010384)
Supplement: S6 Table — Urinary glucose and protein are graded mild, moderate, severe, or potentially life threatening for each lesion count range (lesion count severity score) on admission. Urinary glucose and protein are based on the most severe observation during hospitalization. (DOCX) [file pntd.0010384.s012.docx]

**S6 Table: Urine severity by lesion severity score.**

|  | | **Total Lesion Severity Score** | | | | |  |
| --- | --- | --- | --- | --- | --- | --- | --- |
|  |  | | **<25 (N=20)** | **25-99 (N=58)** | **100-499 (N=91)** | **≥500 (N=47)** | |
| **Laboratory Test** | **Severity** | | **n (%)** | **n (%)** | **n (%)** | **n (%)** | |
| Glucose | Mild | | 1 (5.0) | 2 (3.4) | 1 (1.1) | 0 (0.0) | |
|  | Moderate | | 5 (25.0) | 8 (13.8) | 17 (18.7) | 6 (12.8) | |
|  | Severe | | 0 (0.0) | 0 (0.0) | 1 (1.1) | 0 (0.0) | |
|  | Potentially Life Threatening | | 0 (0.0) | 0 (0.0) | 0 (0.0) | 0 (0.0) | |
|  | | | | | | |  |
| Protein | Mild | | 0 (0.0) | 4 (6.9) | 11 (12.1) | 4 (8.5) | |
|  | Moderate | | 8 (40.0) | 19 (32.8) | 23 (25.3) | 11 (23.4) | |
|  | Severe | | 9 (45.0) | 26 (44.8) | 42 (46.2) | 23 (48.9) | |
|  | Potentially Life Threatening | | 1 (5.0) | 8 (13.8) | 13 (14.3) | 6 (12.8) | |

Laboratory test severity grade based on most severe observation during hospitalization. Total lesion severity score equals total number of lesions present on admission day.
